# DNA methylation by CcrM contributes to genome maintenance in the *Agrobacterium tumefaciens* plant pathogen

**DOI:** 10.1093/nar/gkae757

**Published:** 2024-09-04

**Authors:** Sandra Martin, Florian Fournes, Giovanna Ambrosini, Christian Iseli, Karolina Bojkowska, Julien Marquis, Nicolas Guex, Justine Collier

**Affiliations:** Department of Fundamental Microbiology, Faculty of Biology and Medicine, University of Lausanne, Lausanne CH-1015, Switzerland; Department of Fundamental Microbiology, Faculty of Biology and Medicine, University of Lausanne, Lausanne CH-1015, Switzerland; Bioinformatics Competence Center, University of Lausanne, Lausanne CH-1015, Switzerland; Bioinformatics Competence Center, Ecole Polytechnique Fédérale de Lausanne, Lausanne CH-1015, Switzerland; Bioinformatics Competence Center, University of Lausanne, Lausanne CH-1015, Switzerland; Bioinformatics Competence Center, Ecole Polytechnique Fédérale de Lausanne, Lausanne CH-1015, Switzerland; Lausanne Genomic Technologies Facility, Faculty of Biology and Medicine, University of Lausanne, Lausanne CH-1015, Switzerland; Lausanne Genomic Technologies Facility, Faculty of Biology and Medicine, University of Lausanne, Lausanne CH-1015, Switzerland; Bioinformatics Competence Center, University of Lausanne, Lausanne CH-1015, Switzerland; Bioinformatics Competence Center, Ecole Polytechnique Fédérale de Lausanne, Lausanne CH-1015, Switzerland; Department of Fundamental Microbiology, Faculty of Biology and Medicine, University of Lausanne, Lausanne CH-1015, Switzerland

## Abstract

The cell cycle-regulated DNA methyltransferase CcrM is conserved in most *Alphaproteobacteria*, but its role in bacteria with complex or multicentric genomes remains unexplored. Here, we compare the methylome, the transcriptome and the phenotypes of wild-type and CcrM-depleted *Agrobacterium tumefaciens* cells with a dicentric chromosome with two essential replication origins. We find that DNA methylation has a pleiotropic impact on motility, biofilm formation and viability. Remarkably, CcrM promotes the expression of the *repABC^Ch2^* operon, encoding proteins required for replication initiation/partitioning at *ori2*, and represses *gcrA*, encoding a conserved global cell cycle regulator. Imaging *ori1* and *ori2* in live cells, we show that replication from *ori2* is often delayed in cells with a hypo-methylated genome, while *ori2* over-initiates in cells with a hyper-methylated genome. Further analyses show that GcrA promotes the expression of the RepC^Ch2^ initiator, most likely through the repression of a RepE^Ch2^ anti-sense RNA. Altogether, we propose that replication at *ori1* leads to a transient hemi-methylation and activation of the *gcrA* promoter, allowing *repC^Ch2^* activation by GcrA and contributing to initiation at *ori2*. This study then uncovers a novel and original connection between CcrM-dependent DNA methylation, a conserved epigenetic regulator and genome maintenance in an *Alphaproteobacterial* pathogen.

## Graphical Abstract

**Graphical Abstract:**
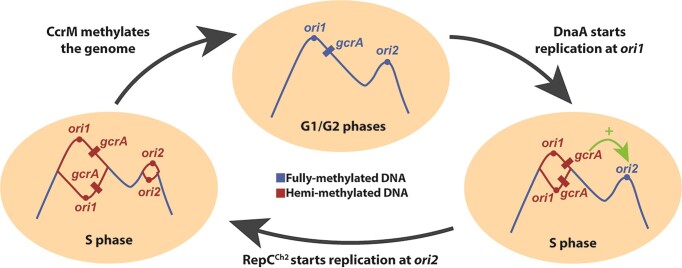

## Introduction

**Figure 1.**
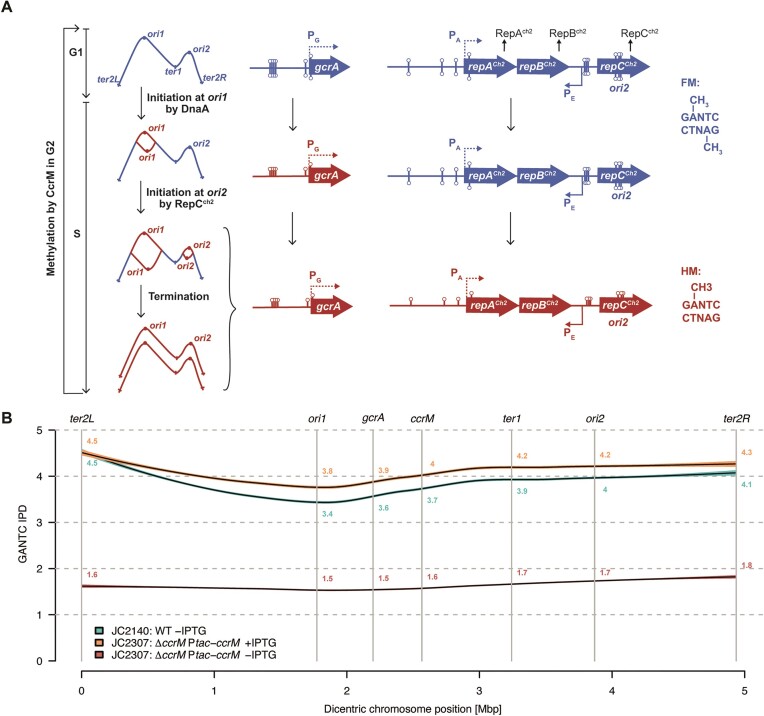
Methylation of GANTC motifs on the dicentric chromosome of *A. tumefaciens*. (**A**) Schematic showing the predicted methylation state of GANTC motifs depending on their chromosomal location and on cell cycle progression. ‘FM’ stands for fully-methylated DNA (blue color) with m6A on both GANTC strands and ‘HM’ stands for hemi-methylated DNA (red color) with m6A on only one GANTC strand (the newly-replicated strand is not yet methylated by the CcrM MTase). The right side of this schematic focuses on the predicted methylation state of the *gcrA* and *repABC^Ch2^* loci as a function of cell cycle progression, which depends on their chromosomal position. Lollipops indicate m6A in GANTC motifs (including 200 bp upstream of ORFs). (**B**) CcrM-dependent methylation of GANTC motifs on the *A. tumefaciens* dicentric chromosome. gDNA samples from WT and JC2307 (*ΔccrM* P*tac-ccrM*) cells that grew exponentially in ATGN ± IPTG for 7 h were analyzed by SMRT-seq. GANTC IPD ratios were directly generated by the PacBio software. A smooth curve was then fitted between these ratios and the dicentric chromosome position using the LOESS function of R with default parameters. The 95% confidence interval was then plotted to predict average GANTC IPD ratios depending on their position on the dicentric chromosome from *ter2L* (position ‘0 Mbp’) to *ter2R* (position ‘4.936 Mbp’).

Approximately 10% of the sequenced bacterial strains display complex genomes with more than one essential replicon and/or more than one essential replication origin. These include many *Alphaproteobacteria* that act as human, animal or plant pathogens, such as *Brucella abortus* or *Agrobacterium tumefaciens*, and that have very diverse modes of life (1,2). Multipartite or multicentric genomes pose significant challenges for genome stability and survival over generations, as the replication and the partitioning of multiple essential origins need to be coordinated with one another and with other events of the cell cycle. Even if ∼26% of the sequenced bacterial strains with multipartite genomes correspond to *Alphaproteobacteria* (3), mechanisms that these bacteria use to ensure the maintenance of such multipartite/multicentric genomes are still largely underexplored compared to *Gammaproteobacteria* (4–6). In *Alphaproteobacteria*, replication of the main chromosome is supposedly always dependent on the very conserved initiator of DNA replication DnaA that usually binds to multiple sites on bacterial origins of replication (7). Instead, replication of secondary chromosomes (carrying essential genes) or mega-plasmids is usually dependent on *repABC* operons (4,5). In such cases, RepC acts as the initiator of DNA replication by binding to an origin that is usually directly located inside the *repC* gene (4,8). Then, RepA and RepB (homologs of ParA and ParB) contribute to replicon partitioning through their binding to *parS* sequences located inside and/or next to the cognate *repABC* operon (9). Interestingly, *repABC* loci usually carry a higher-than-expected number of 5′-GANTC-3′ motifs that are often located in the promoter region driving the transcription of *repABC* operons, in putative origins located inside *repC* genes and in putative *repE* promoter regions driving the transcription of RepE small regulatory RNAs that can down-regulate *repC* transcription and translation (4,10). In nearly all *Alphaproteobacteria* except *Rickettsiales* and *Magnetococcales*, adenines located in such GANTC motifs are methylated (m6A) by the cell cycle-regulated DNA methyltransferase (MTase) CcrM (11). This solitary MTase, which is not associated with a cognate endonuclease, was initially discovered in the *Caulobacter crescentus Alphaproteobacterium* that has a single circular chromosome. In this bacterium, CcrM was shown to be present and active only at the very end of the S-phase of the cell cycle in pre-divisional cells (12,13). As a consequence, newly replicated GANTC motifs spread throughout the genome stay hemi-methylated for a significant period of the cell cycle after the passage of the replication fork, especially if these motifs are located closer to the origin of replication than to the terminus of replication of its unique chromosome (14). In *C. crescentus*, the methylation of hundreds of promoter regions by CcrM has a major impact on its transcriptome (11), notably because the co-conserved global cell cycle regulator GcrA can sense such epigenetic signals to modulate gene expression (15–18). One such gene strongly activated through methylation is the *ftsZ* gene required for cell division (11,19). Molecular genetics and experimental evolution analyses demonstrated that the essentiality of *ccrM* in fast-growing *C. crescentus* is specifically dependent on this key epigenetic activation of *ftsZ* expression (19,20). In *Brevundimonas subvibrioides*, where *ccrM* is not essential, the CcrM and GcrA regulons are significantly different compared to those of *C. crescentus* (21), demonstrating a relatively fast evolution of epigenetic regulatory pathways even in closely related *Alphaproteobacteria*. However, so far, studies did not focus on the impact of CcrM-dependent methylation in *Alphaproteobacteria* with more complex or multipartite genomes, despite observations indicating that CcrM is also essential in *B. abortus* (22) and *A. tumefaciens* (23,24). In the study described here, we aimed at filling this gap using the *A. tumefaciens* plant pathogen, which is the causal agent of the crown gall disease and a live biotechnological tool used for the genetic manipulation of plants (25). Earlier findings indicated that CcrM is also a cell cycle-regulated DNA MTase in this *Alphaproteobacterium* (23). *A. tumefaciens* displays a complex genome with two essential (*ori1* and *ori2*) and two dispensible (*ori^pAt^* and *ori^pTi^*) origins (24,26,27). In the original C58 strain sequenced in 2001, it was shown that its genome consists in one circular chromosome (Ch1 with a DnaA-dependent *ori1*), one linear chromosome (Ch2 with a RepC^Ch2^-dependent *ori2*) and two mega-plasmids encoding important virulence factors (pTi and pAt, with RepC^pTi^/RepC^pAt^-dependent *ori^pTi^* and *ori^pAt^*, respectively) (26,27). However, a very recent study from 2022 showed that many of the C58 strains used in laboratories over the world carry a unique dicentric linear chromosome instead of two distinct chromosomes (Figure 1a, left side) (28). Still, even in such strains, the *repABC^Ch2^* module (including *ori2*) appears to remain essential for survival since it could not be disrupted during Tn-seq experiments and since the *repB^Ch2^* gene could not be deleted (28), showing that initiation at *ori2* and/or *ori2* partitioning are essential in the two described *A. tumefaciens* C58 strains.

Here, we constructed a conditional *ccrM* mutant and combined methylome and transcriptome analyses, together with single cell reporter assays, to test the impact of CcrM-dependent methylation on the expression and the maintenance of the *A. tumefaciens* complex genome. Our results demonstrate that CcrM-dependent methylation is an essential process for fast-growing *A. tumefaciens* cells and that it has a major impact on the global transcriptome including on the expression of its three *repABC* operons. We also found that the timing or the frequency of *ori2* firing/partitioning are affected when the *A. tumefaciens* genome becomes hypo- or hyper-methylated due to genetic perturbations. Altogether, we uncovered an original connection between CcrM-dependent methylation and genome maintenance in a pathogenic *Alphaproteobacterium* with an essential RepABC-dependent replicon.

## Materials and methods

### Bacterial growth conditions

Growth conditions are described in Supplementary Information.

### Bacterial strains, plasmids and oligonucleotides

Bacterial strains, plasmids and primers used in this study are listed in Supplementary Tables S1, S2 and S3, respectively. The genomes of the *A. tumefaciens* C58 derivatives (from (29)) used in this study were analyzed by PCR as described before (28) and this analysis showed that these strains are so-called ‘C58 fusion strains’ with a unique dicentric chromosome (Supplementary Figure S1). This feature was subsequently verified when re-assembling the genomes of the WT/JC2307 strains from whole-genome SMRT-seq data.

### Construction of plasmids and strains

Construction of plasmids and strains is described in Supplementary Information.

### Microscopy

To visualize and analyze cell morphology, cells were fixed using a 5X-fix solution (150 mM NaPO_4_, 12.5% formaldehyde at pH7.5) and stored at 4°C before being imaged on 0.5× PBS (phosphate-buffered saline) and 1% agarose pads using a Plan-Apochromat 100×/1.45 oil phase contrast (Ph3) objective on an AxioImager M1 microscope (Zeiss) with a cascade 1K EMCCD camera (Photometrics) controlled by the VisiView 7.5 software. For phase contrast and fluorescence microscopy, live cells were immobilized onto 0.5× PBS + 1% agarose pads and imaged using the same microscope system as described above. To analyze the subcellular localization and the number of fluorescent origin foci, and to construct demographs, image analyses were performed using the Fiji 2.3.0 software with the MicrobeJ plugin (default parameters) (30).

### gDNA preparation

Genomic DNA samples were prepared from 1 to 2 ml of bacterial cultures. Cells were pelleted and immediately frozen in liquid nitrogen prior to conservation at -80°C. gDNA was extracted using an isopropanol-ethanol purification kit (Puregene Yeast/Bact. Kit B from Qiagen) and following the manufacturer's protocol including a 60-minute RNase A treatment. gDNA was rehydrated in H_2_O. gDNA sample quality and quantity were assessed using a Nanodrop spectrophotometer before storage at −20°C.

### SMRT-sequencing and analyses

High molecular weight gDNA was sheared with the Megaruptor 3 (Diagenode) to obtain 10–15 kb fragments. After shearing, the DNA size distribution was checked using a Fragment Analyzer (Agilent Technologies). Multiplexed SMRTbell libraries were prepared from 365 ng of sheared DNA with the PacBio SMRTbell Express Template Prep Kit 2.0 (Pacific Biosciences) according to the manufacturer's recommendations (protocol 101-696-100, v07). A final size selection step was performed with Pacific Biosciences Ampure beads to remove libraries smaller than 3 kb in size. The resulting libraries were pooled and sequenced over a 15H movie length on a single PacBio Sequel II SMRT cell 8M using the Binding kit version 1.0 and the Sequencing kit version 2.0 (Pacific Biosciences). To analyze the methylome of our strains, the raw data were processed using the SMRTlink version 10.2.0.133434 (Pacific Biosciences, USA). The tool named ‘Base Modification Analysis Application’ was then used to detect modified DNA bases and to identify methylated DNA motifs. IPD values of each GANTC motif reported by the PacBio software along with their genomic position (on the assembled genome sequence of the dicentric C58 strain) were extracted by the Perl script named ‘grabAllMotifs’ and deposited on Zenodo [https://zenodo.org/doi/10.5281/zenodo.12517805]. Additional GANTC motifs present on the genome but absent from the list returned by the PacBio software, were added using the script named ‘completeGANTC’, that also attributed an IPD value of 1.0 to the 3717 (out of 12 680) GANTC motifs from sample JC2307_neg_ipd for which the PacBio software did not return any value or reported ‘NA’. The final resulting IPD file has also been deposited on Zenodo as ‘all_GANTC_fused_WG.txt’, along with the script used to generate Figure 1B.

### RNA extraction

RNA samples were prepared from 4 to 5 ml of bacterial cultures. Cells were pelleted and immediately frozen in liquid nitrogen prior to conservation at −80°C. RNA were extracted using the RNeasy Mini-Kit from Qiagen following the manufacturer's protocol and including a DNase I (RNase-free DNases set from Qiagen) treatment. Samples were additionally treated with a TURBO DNA-free kit from Invitrogen following the manufacturer's protocol. RNA samples were purified again and eluted in water. Absence of DNA contaminations was verified by standard PCR. Quality and quantity of RNA samples were verified on an agarose gel and using a Nanodrop spectrophotometer before storage at −80°C.

### RNA-sequencing and analyses

RNA quality was assessed using a Fragment Analyzer (Agilent Technologies) and all RNAs had RQNs between 9.7 and 10. RNA-seq libraries were prepared from 800 ng of total RNA with the TruSeq Stranded mRNA Prep reagents (Illumina) using a unique dual indexing strategy, and following the official protocol automated on the Sciclone liquid handling robot (PerkinElmer). The polyA selection step was replaced by an rRNA depletion step with the QIAseq FastSelect - 5S/16S/23S bacterial rRNA removal kit (Qiagen). Libraries were quantified by a fluorometric method (QubIT, Life Technologies) and their quality assessed using the Fragment Analyzer. Sequencing was performed on the Illumina HiSeq 4000 v4 SR flow cell with the v4 HiSeq 3000/4000 SBS Kit reagents for 150 cycles. Sequencing data were demultiplexed using the bcl2fastq2 Conversion Software (version 2.20, Illumina). Sequences matching to ribosomal RNA sequences were removed with fastq_screen (v. 0.11.1) (31). Remaining reads were further filtered for low complexity with reaper (v. 15–065) (32). Reads were aligned against the *Agrobacterium fabrum C58 ASM9202v1* genome using STAR (33) (v. 2.5.3a). The number of read counts per gene locus was summarized with htseq-count (34) (v. 0.9.1) using a custom *Agrobacterium fabrum C58 ASM9202v1* gene annotation. Quality of the RNA-seq data alignment was assessed using RSeQC (35) (v. 2.3.7). Counts per gene table was used for statistical analysis in R (R version 4.1.0). Genes with low counts were filtered out according to the rule of 1 count per million (cpm) in at least 1 sample. Library sizes were scaled using TMM normalization (EdgeR package (36) version 3.34.0) and log-transformed with limma cpm function (Limma package (37) version 3.48.0). Differential expression was computed with limma by fitting the samples into a linear model and performing comparisons with moderated t-test. Global p-value adjustment with Bonferroni–Hotchberg method was used for all comparisons. Genes with an adjusted *P*-value < 0.01 and a minimum fold-change of 2 were considered as significantly mis-regulated. Volcano plots showing differentially expressed genes were made in R (v4.2.1) by plotting the log2(fold change) against the –log_2_(adjusted *P*-value) for this comparison.

### qRT-PCR experiments and analyses

Primers were chosen based on secondary structure predictions using the Mfold web server for nucleic acid folding and hybridization prediction (default parameters, except ‘folding temperature’ set to 60°C) (38). Primer pairs were then checked by standard PCR on *A. tumefaciens* C58 gDNA (1 ng/μl) to verify that they gave a single amplification product of the expected size. Primer pairs were then checked again by qPCR (4 times 4-fold serial dilutions) to check efficiency. 700 ng of RNA samples were retrotranscribed using the Verso cDNA synthesis kit (Thermoscientific) for 30 min at 42°C into a final volume of 20 μl and following the manufacturer's protocol. The Verso reverse transcriptase was then inactivated by a 2-min incubation at 95°C before cDNA samples were stored at –20°C. For each qPCR assay, 2.4 μl of cDNA samples diluted 1:20 in Tris 10mM (pH 8.5) were used as templates. The qPCR reaction mix contained 0.3 μM of each primer into 10 μl of Power SYBR Green PCR Master Mix (Applied Biosystems). The 384-well plate was filled using an Evo® TECAN robot and transferred into a qPCR machine (Thermofisher - QuantStudio6) with automated threshold calculations (Software QuantStudio Q6_v1.6). Cycling: 10 min at 95°C once, 15 s at 95°C and 1 min at 60°C 40 times, and then 15 s at 95°C once. For RNA samples from the JC2307 strain, differences in gene expression were evaluated based on stable internal gene controls chosen from the RNAseq data: *purH (Atu_2823*, bifunctional purine biosynthesis protein) for ATGN ± IPTG 7 h conditions or *yidC (Atu_0384*, membrane protein insertase YidC) for YEB ± IPTG 5.5 h conditions. For RNA samples from the JC2899 strain, differences in gene expression were evaluated based on the stable *hemF* (*Atu_2247*, oxygen-dependent coproporphyrinogen oxidase) internal gene control. The Delta-Delta Ct method (39) was used to estimate relative mRNA levels from averages calculated from three technical replicates. Three biological replicates were used for each strain/condition tested.

### Conjugation assays for functional analysis of the RepABC^Ch2^/*ori2* module

pSW25T-repABC^Ch2^ derivatives were transferred from MFDpir *E. coli* donor cells (DAP auxotrophs in which the R6K origin is functional) to recipient *S. meliloti* Rm1021 cells (Strep^R^ cells in which the R6K origin is not functional) by conjugation to determine if these plasmids were able to replicate in this *Alphaproteobacterium*. *S. meliloti* Rm1021 cells were grown overnight in LB supplemented with streptomycin, while MFDpir *E. coli* cells containing the pSW25T-repABC^Ch2^ derivative were grown overnight in LB supplemented with DAP and kanamycin. The two overnight cultures (250mL) were then centrifuged at 8000 rpm for 3 min, washed and resuspended into 50 mL of LB supplemented with DAP. Next, donor and recipient cells were mixed 50/50 and spotted onto a nitrocellulose filter membrane placed on top of an LB agar plate supplemented with DAP. Plates were incubated overnight at 30°C. The next morning, cells were resuspended into 2ml of LB. To determine the proportion of *S. meliloti* Rm1021 cells that acquired the pSW25T-repABC^Ch2^ derivative (*trans*-conjugants), 100 μl aliquots from serial 10-fold dilutions were plated on selective LB plates supplemented with kanamycin and streptomycin and on non-selective LB plates supplemented with streptomycin alone. After three days of growth at 30°C, conjugation efficiencies were estimated by calculating the ratio between the number of *trans*-conjugants (colonies that grew on selective media) and the total number of potential recipient cells (colonies that grew on non-selective media).

### Promoter activity measurements by fluorometry

pOT1e derivatives (with WT or mutant P_A_-*egfp* or P_E_-e*gfp* fusions) were inserted into *C. crescentus* NA1000 (JC450) cells by transformation. The resulting transformants were first grown overnight at 28°C in PYE supplemented with gentamycin and then diluted back into M2G supplemented with gentamycin. Overnight cultures were then diluted to an OD_600_∼0.05 into the same medium and transferred into 96-well-plates. Fluorescence intensities and OD_600_ were measured after ∼2 generations of growth (OD_600_∼ 0.2 ± 0.05) using a microplate reader (Biotek Synergy H1) using the following parameters: excitation at 488 nm and emission at 510 nm. Relative fluorescence units (RFU) were calculated as the fluorescence intensities (AU)/OD_600._ Promoter activities were then estimated by calculating the ratio between the RFU of test strains (carrying pOT1e derivatives with cloned promoters) and the RFU of the control strain with the empty pOT1e.

### Statistics and reproducibility

Statistical methods and sample sizes (*n*) are indicated in figure legends for each experiment. Statistical analyses were done using the Excel, GraphPad-PRISM or R softwares.

## Results

### CcrM is essential in *A. tumefaciens* cells cultivated in complex media

**Figure 2.**
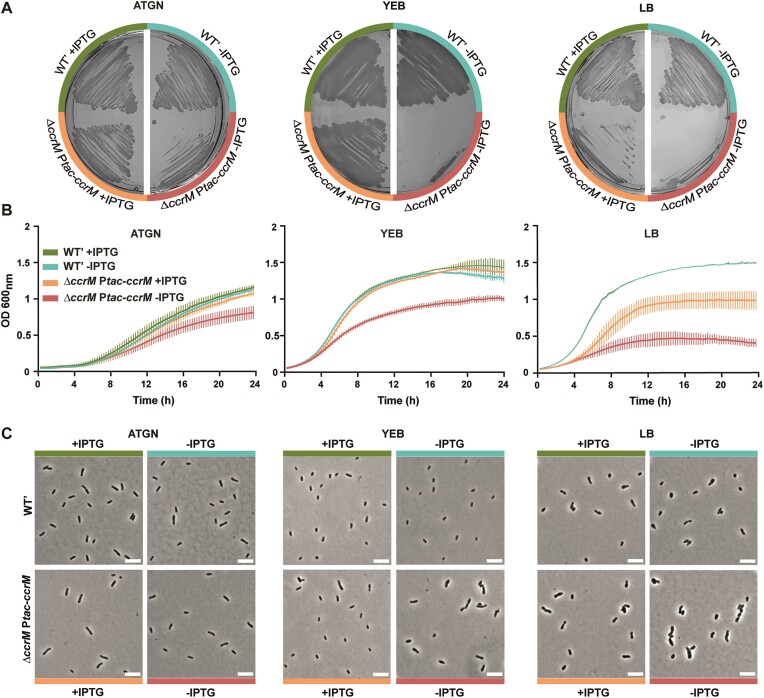
Depletion of CcrM results in slow growing, elongated and swollen cells when cultivated in complex media. (**A**) JC2141 **(**WT’) and JC2307 (*ΔccrM* P*tac-ccrM*) cells were plated onto ATGN, YEB or LB agarose plates containing or not the IPTG inducer and colonies were then cultivated for ∼3 days prior to imaging. (**B**) JC2141 and JC2307 strains were pre-cultured into liquid ATGN, YEB or LB media + IPTG. Cells from over-night cultures were then washed and diluted to an OD_600_∼0.1 into the indicated medium and placed into a plate reader to record the OD_600_ every 15 min. Error bars represent means ± standard deviations from three biologically independent replicates. Note that the two WT’ curves overlap for the LB growth condition. (**C**) JC2141 and JC2307 strains were pre-cultured into liquid ATGN + IPTG and then diluted into ATGN or YEB or LB with IPTG. Once cultures reached exponential phase again, cultures were washed and cells were resuspended to an OD_600_∼0.2 into ATGN, YEB or LB ± IPTG at time T0. Cells were then fixed and imaged by phase-contrast microscopy after 24 h of growth. White scale bars inside images are 5 μm.

Considering the predicted essentiality of *ccrM* in *A. tumefaciens* (23,24), we engineered a conditional *ccrM* mutant to be able to analyze the methylome, the transcriptome and the phenotypes of cells as CcrM gets depleted. This enabled us to obtain information on the impact of DNA methylation in *A. tumefaciens*. In practice, a second copy of *ccrM* (*Atu0794*) under the control of the IPTG-inducible P*tac* promoter was introduced at the *tetRA* locus of the dicentric chromosome of an *A. tumefaciens* C58 derivative strain, and then the native *ccrM* gene was deleted following a standard double recombination procedure (29), generating strain JC2307 (*ΔccrM* P*tac-ccrM*) on ATGN minimal medium containing IPTG. Even if colonies grew very slowly on ATGN lacking IPTG compared to ATGN with IPTG (Figure 2A), colonies were still largely detectable, indicating either that CcrM was not efficiently depleted in the absence of the IPTG inducer, or that *ccrM* is not strictly essential under such growth conditions. Consistent with this first observation, we also found that JC2307 cells could grow relatively well in liquid ATGN medium, even if their growth rate progressively decreased over time after the removal of the IPTG inducer (Figure 2B). Colony forming unit (CFU) assays also showed that cell viability remained normal for extended periods of time (Supplementary Figure S2b, c), even if CcrM levels already became very hard to detect from cell extracts using immunoblotting experiments after 7 h of growth in ATGN without IPTG (Supplementary Figure S3). The morphology of CcrM-depleted cells (cultivated in ATGN without IPTG) appeared as relatively similar to control cells (Figure 2C), even if quantitative analyses showed that such cells were slightly shorter than control cells expressing *ccrM* (median length of 1.64 μm compared to 1.94–1.99 μm) (Supplementary Figure S4, ATGN).

Strikingly, the impact of CcrM depletion was much stronger in cells cultivated in complex media. Indeed, JC2307 cells could hardly form colonies on YEB or LB plates lacking IPTG (Figure 2A), grew very slowly after a few generations in liquid YEB or LB media without IPTG (Figure 2B) and appeared as significantly elongated and/or enlarged (Figure 2C and Supplementary Figure S4) before they started to lyse/die (Supplementary Figure S2b, c). Time-lapse microscopy experiments also showed that most JC2307 cells cultivated onto YEB agarose pads lacking IPTG lysed or developed abnormal morphologies (swollen, elongated and multi-polar cells) over time (Supplementary Figure S5 and Supplementary Movies S1-S3). Importantly, the presence of IPTG in these media to induce P*tac-ccrM* led to a complete (in YEB) or partial (in LB) complementation of these phenotypes (Figure 2 and Supplementary Figure S2 and S4), showing that they were, indeed, connected to insufficient levels of CcrM and not to polar effects.

Altogether, the use of this conditional mutant for phenotypic analyses demonstrated that CcrM is essential for the survival of *A. tumefaciens* cells cultivated in or on complex media, but that it may be dispensable, or at least not required at similar levels, in cells cultivated in or on minimal media.

### CcrM-depleted cells cultivated in minimal medium have a hypo-methylated genome and display motility and adhesion defects

To test if CcrM levels become too limiting to ensure an efficient methylation of genomic GANTC motifs in the conditional mutant cultivated in minimal medium (when cells remain viable), we performed two assays. First, we compared the efficiency of digestion by HinfI of gDNA samples prepared from WT or JC2307 cells cultivated in ATGN with or without IPTG. HinfI is a methylation-dependent endonuclease that can only cut non-methylated GANTC motifs (23). Consistent with the known efficient activity of CcrM in *A. tumefaciens*, we confirmed that restriction of the WT genome by HinfI was essentially undetectable (Supplementary Figure S6). In contrast, the genome of JC2307 cells cultivated in ATGN without IPTG for 7 h became highly sensitive to HinfI digestion (Supplementary Figure S6), indicating that a majority of its double-stranded GANTC motifs become un-methylated when CcrM is depleted. To get a more quantitative evaluation of the methylation state of the *A.tumefaciens* genome upon CcrM depletion, we next analyzed the methylome of cells cultivated in these same growth conditions using single molecule real-time sequencing (SMRT-Seq), a method that is now commonly used to distinguish methylated (m6A) from non-methylated adenines on bacterial genomes based on measures of interpulse duration (IPD) (40,41). Using gDNA extracted from WT cells, we found that the average IPD ratio of adenines located in GANTC motifs located near *ori1* (IPD ∼ 3.4) was lower than that of GANTC motifs located next to *ter2L* (IPD ∼ 4.5) on the dicentric chromosome (Figure 1B), which is fully consistent with the known cell cycle regulation of CcrM-dependent methylation in *A. tumefaciens* cells (Figure 1A) (23). Indeed, if CcrM was present and active during the whole S-phase of the cell cycle in *A. tumefaciens*, newly replicated double-stranded GANTC motifs located next to *ori1* would not stay in a hemi-methylated state for a long enough period to be detectable. Interestingly, the methylome of JC2307 (*ΔccrM* P*tac-ccrM*) cells cultivated in ATGN with the IPTG inducer was very similar to the methylome of WT cells (Figure 1B and Supplementary Figure S7), indicating that CcrM is probably regulated by post-transcriptional mechanisms of regulation in *A. tumefaciens*, similarly to what happens in *C. crescentus* (42,43). In contrast, using gDNA from JC2307 cells cultivated in ATGN without IPTG for 7 h, the average IPD ratio of adenines located in GANTC motifs throughout the dicentric chromosome dropped to an average value of ∼1.6 (compared to an average of ∼3.8 for WT cells and ∼4.1 for *ΔccrM* P*tac-ccrM* cells cultivated in ATGN with IPTG) (Figure 1B and Supplementary Figure S7). This observation confirmed that a vast majority of the 5166 double-stranded GANTC motifs found on the dicentric chromosome of JC2307 cells are in a non-methylated state after 7 hours of CcrM depletion. Importantly, finding viable conditions (Figure 2 and Supplementary Figure S2) when *A. tumefaciens* cells display a largely hypo-methylated genome (Figure 1B and Figures S6 and S7) made it possible to test the impact of DNA methylation by CcrM on the transcriptome of *A. tumefaciens* cells with a complex multicentric genome.

Interestingly, even if such mutant cells did not display striking viability or cell morphology defects in ATGN medium (Figure 2 and Figures S2 and S4), we still noticed two interesting phenotypes. First, cells displayed a significant swarming defect as could be seen with the ∼35% decrease in swarming area when placed onto semi-solid ATGN medium without IPTG compared to semi-solid ATGN with IPTG (Supplementary Figure S8a). Second, we observed a ∼35% decrease in adhesion/biofilm formation in ATGN without IPTG (Supplementary Figure S8b) as could be measured using static coverslip assays with crystal violet (29). These phenotypes may relate to the slower growth of CcrM-depleted cells or result from changes in gene expression.

Altogether, these methylome and phenotypic assays showed that *A. tumefaciens* cells with hypo-methylated GANTC motifs on their genome are viable in minimal medium, but still display detectable phenotypes suggesting that some genes may be mis-expressed as a result of their hypo-methylation.

### DNA methylation by CcrM has a major impact on the *A. tumefaciens* transcriptome

**Figure 3.**
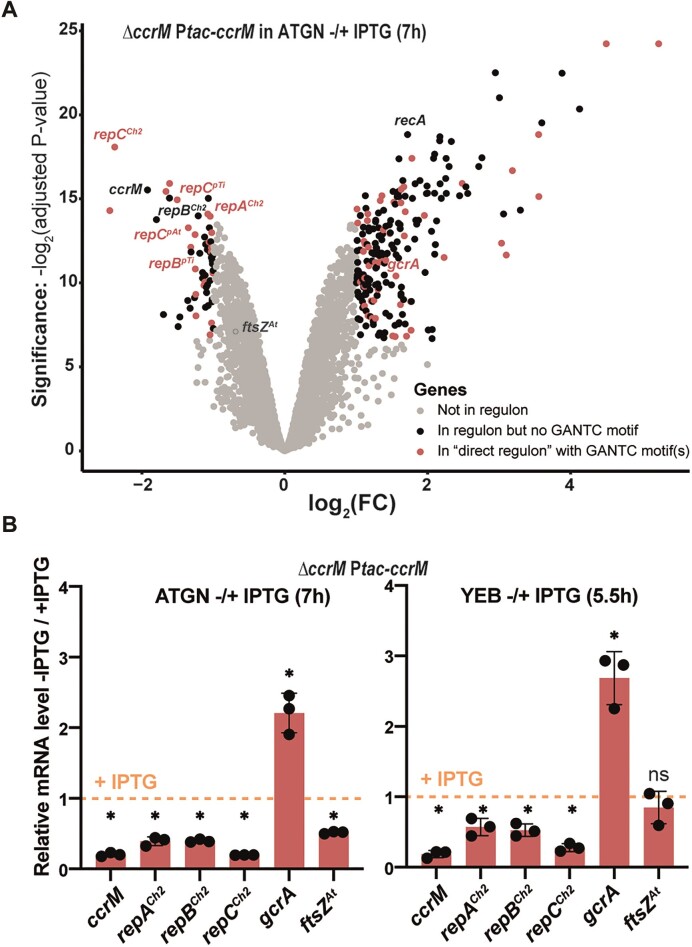
Depletion of CcrM has a major impact on the *A. tumefaciens* transcriptome in cells cultivated for 7 hours in minimal medium. (**A**) Volcano plot showing RNA-Seq results and analyses comparing the transcriptome of exponentially growing JC2307 (Δ*ccrM* P*tac-ccrM)* cells cultivated in ATGN ± IPTG for 7 h (same cultures as those used for methylome experiments described in Figure 1B). FC indicates the fold-change when comparing the - IPTG condition with the + IPTG condition. Each dot corresponds to a gene. Grey dots correspond to genes that are not considered as differentially regulated (= FC < 2 or adjusted *P*-value > 0.01). Black dots correspond to 198 genes that are differentially regulated (FC > 2 and adjusted *P*-value < 0.01) but that do not contain a GANTC motif in the 200 bp region upstream of the ORF. Red dots correspond to the 75 genes that are differentially regulated (FC > 2 and adjusted *P*-value < 0.01) and that do contain minimum one GANTC motif in the 200 bp region upstream of the ORF (‘direct regulon’). The precise identity/annotation/COG category/GANTC motif locations for all genes can be found in Supplementary Tables S4 and S5. A Glimma Volcano Plot (interactive HTML graphic) is also available in the supplementary information. Adjusted *P*-values were calculated based on three independent biological replicates for each strain/growth condition. (**B**) qRT-PCR results confirming the impact of CcrM on the expression of a selection of genes in JC2307 (Δ*ccrM* P*tac-ccrM*) cells cultivated in minimal (left) and complex (right) media. The graphs show relative mRNA levels in cells cultivated without the IPTG inducer, compared to cells cultivated with the IPTG inducer (then set to an arbitrary value of 1 for each gene = orange dotted line). The same RNA samples as those used in (A) were used for the ‘ATGN ± IPTG (7 h)’ panel. RNA samples used for the ‘YEB ± IPTG (5.5 h)’ panel were prepared from JC2307 cells pre-cultured in ATGN + IPTG and then diluted into YEB + IPTG for growth until exponential phase. Cells were then washed and resuspended into YEB ± IPTG at Time 0. RNA samples were prepared after 5.5 h of growth. For both panels, three biological replicates with three technical replicates each were used. Significant differences (Wilcoxon rank sum test) when comparing – IPTG / + IPTG are indicated by * (*P*< 0.0001). ns: not significant (*P*-value > 0.05).

To collect information on the origin of the observed phenotypes (Figure 2 and Supplementary Figure S2, S4 and S8) and gather potential cues on why DNA methylation by CcrM becomes essential in cells cultivated in complex media (Figure 2 and Supplementary Figure S2), we next decided to compare the transcriptome of viable *A. tumefaciens* JC2307 cells after the drop in detection of methylated GANTC motifs (7 h in ATGN without IPTG) with that of mutant cells cultivated in ATGN with IPTG or of WT cells (Figure 1B). RNA-Seq experiments revealed a significant (adjusted *P*-value < 0.01) and strong (fold change > 2) impact on the expression of 273 genes (Figure 3A, Supplementary Table S4 and Supplementary Figure S9): 59 genes were down-regulated, while 214 genes were up-regulated in response to CcrM depletion (comparing JC2307 cells cultivated with or without IPTG for 7 h). Instead, the transcriptome of CcrM-repleted cells (JC2307 cells cultivated with IPTG), appeared as extremely similar to the transcriptome of WT cells (Supplementary Figure S9 and Supplementary Table S4), showing that expression of *ccrM* from the P*tac* promoter instead of from its native promoter does not lead to significant gene mis-regulation. Among the 273 genes that were significantly mis-regulated upon CcrM depletion, 75 genes (27%) displayed at least one GANTC motif in their putative promoter region (200 bp upstream of each ORF), including 22 of the 59 (37%) down-regulated genes and 53 of the 214 (25%) up-regulated genes (Figure 3A and Supplementary Table S5). Thus, genes with putative promoters carrying GANTC motif(s) are significantly enriched among the genes that appear as (directly or indirectly) activated by CcrM compared to random *A. tumefaciens* genes (24% have GANTC motif(s) in their putative promoter region).

As a first point of interest, we compared this potential ‘direct regulon’ of CcrM (75 genes) in *A. tumefaciens* (Supplementary Table S5) with previously published ‘direct regulons’ of CcrM in the distantly related *C. crescentus* (152 genes) and *B. subvibrioides* (129 genes) *Alphaproteobacteria*. This analysis showed that only six genes of the *A. tumefaciens* CcrM ‘direct regulon’ had orthologs that also belonged to the *C. crescentus* or *B. subvibriodes* ‘direct regulons’ (Supplementary Table S5). Even if the precise transcriptional start site (TSS) of each gene/operon is not yet known in *A. tumefaciens*, we concluded that epigenetic mechanisms of regulation apparently evolved significantly between *Alphaproteobacteria* with different genome architectures and lifestyles.

Another striking new finding was that the *A. tumefaciens* CcrM regulon included six genes belonging to the three *repABC* operons (essential for the replication of the dicentric chromosome and of the two mega-plasmids) among the genes that were significantly down-regulated in CcrM depleted cells (Figure 3a and Supplementary Table S5). In addition, many genes encoding proteins potentially involved in SOS-related responses to DNA damage (44) or replication defects (examples: RecA, RecQ, three orthologs of DNA Pol Y and three orthologs of ImuA) were significantly up-regulated. Noteworthy, the *gcrA* homolog of *A. tumefaciens (Atu0426)*, encoding a putative methylation-sensitive global regulator, was strongly activated (2.8-fold induction) upon CcrM depletion, while the *ftsZ^At^* (*Atu2086/ftsZ2*) gene required for *A. tumefaciens* cell division was hardly repressed (less than 2-fold) in ATGN medium (Figure 3 and Supplementary Table S4) confirming strong differences with what was previously observed in *C. crescentusΔccrM* mutant cells (11,19). The impact of CcrM depletion on the expression of this interesting selection of genes was also verified by qRT-PCR (Figure 3B and Supplementary Figure S10) using RNA samples prepared not only from mutant cells cultivated in ATGN ± IPTG for 7 h (same conditions as the RNA-Seq experiments), but also from mutant cells cultivated in YEB ± IPTG for 5.5 h (prior to the detection of cell death without the IPTG inducer of P*tac-ccrM* as shown in Supplementary Figure S2). These experiments showed that a lack of DNA methylation by CcrM also reduces *repC^Ch2^/repC^pTi^/RepC^pAt^* expression and promotes *gcrA* expression in cells cultivated in complex YEB medium, while it does not have a significant impact on the expression of the essential *ftsZ^At^*gene under such growth conditions (Figure 3B, right panel).

These discoveries led us to hypothesize that CcrM-depleted cells may express sub-optimal levels of the essential RepC^Ch2^*ori2* initiator and of the RepAB^Ch2^*ori2* partitioning proteins, leading to potential replication delays from *ori2*. This may also relate to the essentiality of *ccrM* in fast-growing *A. tumefaciens* cells (in YEB and LB media) (Figure 2 and Supplementary Figure S2) since initiation at *ori2* is apparently essential in *A. tumefaciens* (28).

### Replication/partitioning of *ori2* is decoupled from replication/partitioning of *ori1* in CcrM-depleted cells

**Figure 4.**
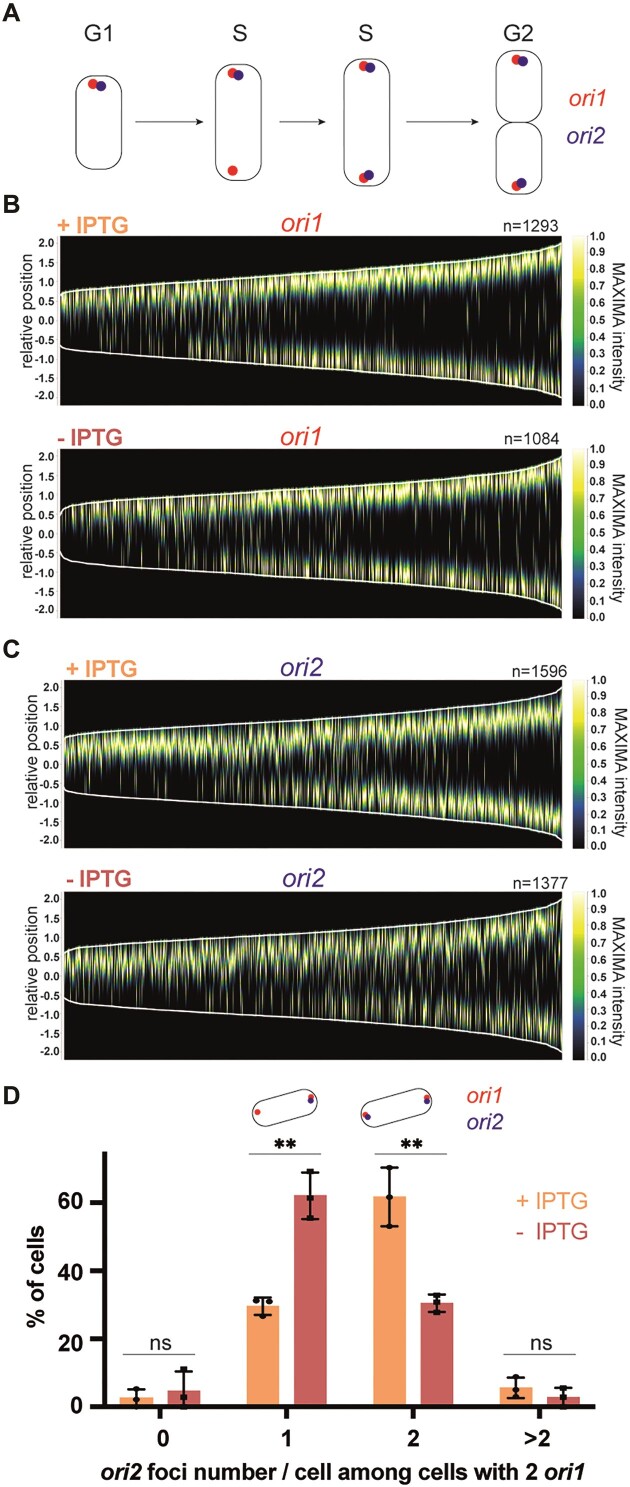
Depletion of CcrM results in *ori2* replication/partitioning delays in cells cultivated in minimal medium for prolonged periods of time. (**A**) Schematic representing the subcellular localization of *ori1* and *ori2* during the *A. tumefaciens* cell cycle. (**B** and **C**) Demographs showing the subcellular localization of *ori1* (B) or *ori2* (C) foci as a function of cell size (from images as shown in Supplementary Figure S8). JC2660 (*ΔccrM* P*tac-ccrM* with *ori1/ygfp* reporter in (B)) or JC2661 (*ΔccrM* P*tac-ccrM* with *ori2/ygfp* reporter in (C)) cells were cultivated over-night in ATGN ± IPTG. Cultures were then diluted into ATGN ± IPTG and grown exponentially for ∼6.5 h (same medium at all steps). Relative position = 0 corresponds to mid-cell; only cells measuring from 1 to 4 μm-long were included into these demographs; *n* = number of cells used to construct each demograph. (**D**) Quantification of *ori2* number per cell among cells that are in S-phase (with two *ori1*). JC2836 (*ΔccrM* P*tac-ccrM* with *ori1/mcherry* and *ori2/ygfp* reporters) cells were cultivated over-night in ATGN ± IPTG. Cultures were then diluted into ATGN ± IPTG and grown exponentially for ∼6.5 h (>15 h into ATGN ± IPTG). Phase contrast/YGFP/mCherry images were acquired as shown in Supplementary Figure S9a. Among cells that displayed minimum two *ori1* foci (S-phase cells from Supplementary Figure S10), the number of *ori2*/cell was measured (from minimum 100 cells/condition) and means from three independent experiments were plotted for each condition. Error bars correspond to standard deviations. Student's *t*-test: ns = *P*-value > 0.01, ** = *P*-value < 0.01.

A few recent studies used live cell fluorescence microscopy experiments to visualize the number and the sub-cellular localization of *ori1* and *ori2* as a function of cell cycle progression (cell length). These studies showed that *ori1* and *ori2* colocalize at the old pole of newborn G1 phase cells (45) (Figure 4A). At the onset of the S-phase of the cell cycle, replication first starts at *ori1* and then at *ori2* following a significant delay. Once duplicated, one copy of each origin (*ori1* and then *ori2*) moves towards the new cell pole (46) (Figure 4A). Importantly, the timing of origin firing and the localization of origins are strikingly similar in C58 strains with two chromosomes compared with strains with one dicentric chromosome (28). We therefore decided to use these same fluorescent reporters to visualize the impact of CcrM depletion on the timing of *ori1* and *ori2* replication and on their partitioning during the *A. tumefaciens* cell cycle.

As a first test, we visualized *ori1* or *ori2* localization using a *ygfp-parB^pMT1^-parS^pMT1^* reporter integrated next to *ori1* or *ori2* (46), respectively, in the dicentric chromosome of JC2307 (*ΔccrM* P*tac-ccrM*) cells. Cells were cultivated exponentially in ATGN ± IPTG and then imaged by fluorescence microscopy. Analyses of images (Supplementary Figure S11a and b) and demographs (Figure 4B, C) revealed that the duplication/partitioning of *ori2* tends to take place in CcrM-depleted (ATGN - IPTG) cells that have reached a longer length compared to CcrM-repleted (ATGN + IPTG) cells (Figure 4C), while no obvious difference could be seen when looking at *ori1* duplication/partitioning (Figure 4B). Moreover, a lower proportion of CcrM-depleted cells (∼18%) displayed two *ori2* foci compared to CcrM-repleted cells (∼38%) (Supplementary Figure S11b), while CcrM depletion had an insignificant impact on the overall proportion of S-phase cells (∼39% instead of 43% of cells displayed 2 *ori1* foci) (Supplementary Figure S11a). These first observations suggested that *ori2* duplication or partitioning is/are specifically delayed in cells that display a hypo-methylated genome.

As a second test, we also co-visualized *ori1* and *ori2* in JC2307 cells using a first *ygfp-parB^pMT1^-parS^pMT1^*reporter near *ori2* and a second *mcherry-parB^P1^-parS^P1^* reporter near *ori1* (45). Fluorescence imaging of cells growing exponentially in ATGN ± IPTG (Supplementary Figure S12a) and analyses of demographs (Supplementary Figure S12b) confirmed that duplication/partitioning of *ori2* tends to take place in CcrM-depleted (ATGN - IPTG) cells that have reached a longer length compared to CcrM-repleted (ATGN + IPTG) cells. Quantitative analyses using this double-labelled strain further showed that the proportion of S-phase cells (with two *ori1* foci) also displaying two *ori2* foci dropped ∼2-fold (from ∼62% to ∼30%) when mutant cells were cultivated in ATGN-IPTG compared to ATGN + IPTG (Figure 4D), while the overall proportion of S-phase cells (with two *ori1* foci) remained essentially similar (from ∼58% to ∼52%) (Supplementary Figure S13). Altogether, these results indicate that replication initiation at *ori2* and/or *ori2* partitioning are/is specifically delayed in mutant *A. tumefaciens* cells displaying a hypo-methylated genome, coinciding with a lower expression of the *repABC^Ch2^* operon (Figure 3).

### Chromosome replication over-initiates from *ori2* in CcrM-overexpressing cells

**Figure 5.**
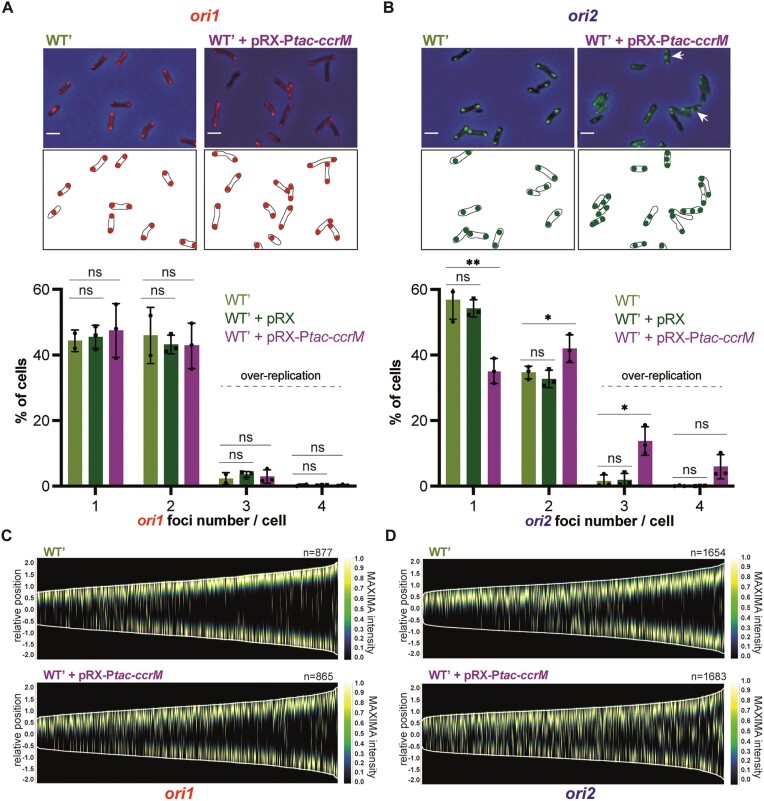
Overexpression of CcrM results in hyper-initiation events at *ori2* in cells cultivated in minimal medium. (**A**) and (**B**) selected microscopy images of JC2656 (WT' with *ori1/ygfp* reporter) cells (panel A) or JC2657 (WT' with *ori2/ygfp* reporter) carrying or not the pRX-P*tac-ccrM* vector. Cells were cultivated over-night in ATGN + IPTG. Cultures were then diluted into ATGN + IPTG and grown exponentially for ∼6.5 h. Upper panels: overlays of phase contrast and YGFP images. Lower panels: schematics showing *ori1* (red color in (A)) or *ori2* (green color in (B)) subcellular localization in the cells imaged above. Under these microscopy images, plots show the proportion of cells with the indicated number of *ori1* (in (A)) or *ori2* (in (B)) foci for each strain (pRX is the empty control vector). Minimum two independent experiments (with a total of minimum 1000 cells) were done for each strain with each dot corresponding to an independent experiment and mean values were plotted with error bars corresponding to standard deviations. Student's *t*-test: ns = *P*-value > 0.05, * = *P*-value < 0.05, ** = *P*-value < 0.01. (**C**, **D**) Demographs showing the subcellular localization of *ori1* (panel c) or *ori2* (panel d) foci as a function of cell size (from images as shown in panels (A) and (B)). Relative position = 0 corresponds to mid-cell; only cells measuring from 1 to 4 μm-long were included into these demographs; n = number of cells used to construct each demograph.

The results described above suggest that DNA methylation by CcrM promotes DNA replication from *ori2* in *A. tumefaciens* cells. To confirm this hypothesis, we also looked at the impact of CcrM over-expression on the number of *ori1* and *ori2* foci per cell. We expect that CcrM over-expression from the pRX-P*tac-ccrM* vector leads to a significant hyper-methylation of the *A. tumefaciens* genome with most GANTC motifs being fully-methylated throughout the cell cycle as previously shown using related vectors (23). In ATGN medium containing IPTG, only ∼35% of the cells over-expressing CcrM displayed a single *ori2* focus, compared to ∼54% of the cells carrying the empty control vector, while differences concerning *ori1* appeared as non-significant (47% compared to 45% with *P*-value > 0.05) (Figure 5A, B). This observation suggested that replication from *ori2* can start sooner after (or even before) replication from *ori1* in cells with a hyper-methylated genome compared to WT cells, which is supported by demographs comparing the timing of *ori1*/*ori2* duplication as a function of cell size/cell cycle progression (Figure 5C, D). Consistent with such a boost of replication from *ori2*, we also observed that ∼20% of the CcrM-overexpressing cells displayed more than two *ori2* foci per cell, something that only very rarely (∼2% of cells) happened in WT cells (Figure 5A, B). Thus, *A. tumefaciens* cells with a hyper-methylated genome regularly over-initiate DNA replication from *ori2*.

**Figure 6.**
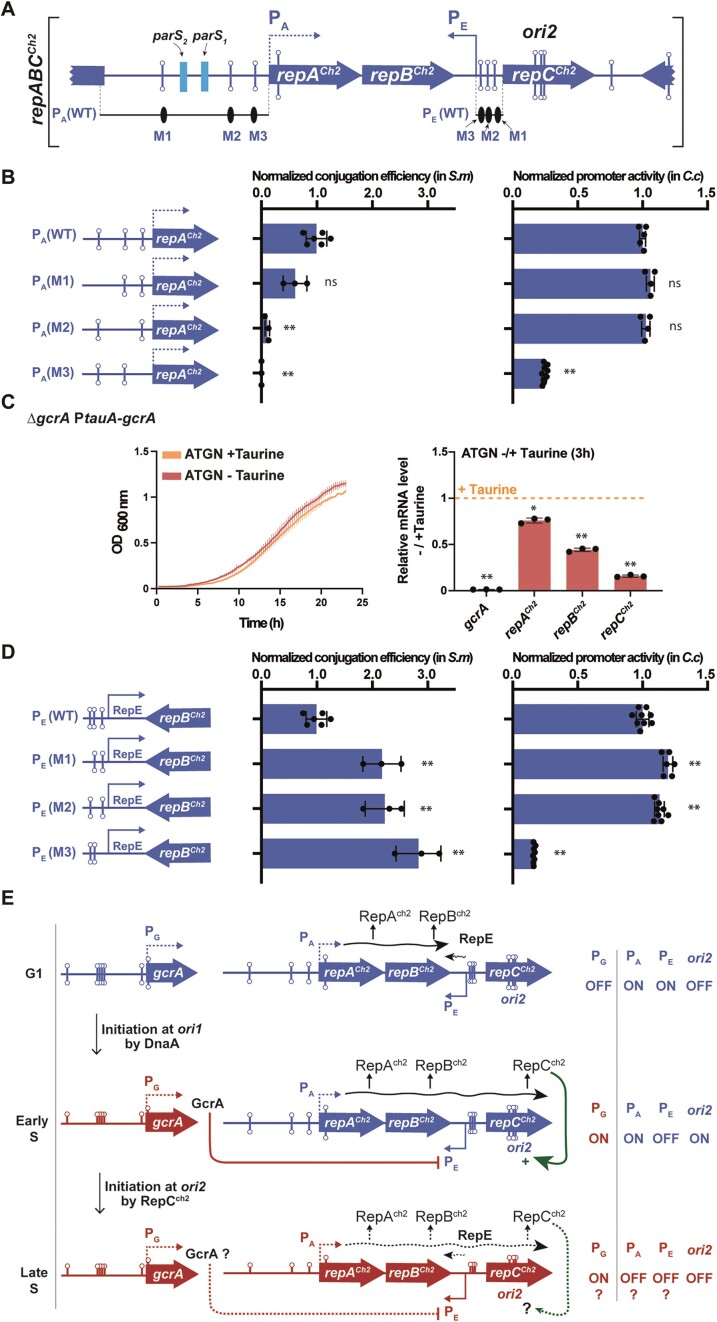
The RepABC^Ch2^/*ori2* module is sufficient for autonomous replication and the expression of its RepC^Ch2^ initiator is activated by GcrA. (**A**) Schematic of the *repABC^Ch2^/ori2* module showing where ORFs (blue arrows), GANTC motifs (lollipops) and predicted *parS* sites (light blue rectangles) (9) are located. Note that the three ORFs are not drawn to scale to better visualize the organization of IG regions. This schematic shows the exact WT module cloned into pSW25T for the conjugation assays shown in the left panels of (B) and (D). It also shows the P_A_(WT) and P_E_(WT) promoter regions cloned into pOT1e for the promoter activity assays shown in the right panels of (b) and (d), respectively. (**B**) Impact of the three GANTC motifs found in the P_A_(WT) promoter on plasmid replication/maintenance in *S. meliloti* cells (left) and on promoter activity in *C. crescentus* cells (right). For the P_A_(M1), P_A_(M2) and P_A_(M3) derivatives of P_A_(WT), single methylatable GANTC motifs were mutated into non-methylatable GTNTC motifs. Means from minimum 3 independent experiments were plotted for each construct. (**C**) The impact of GcrA on *repABC^Ch2^* expression in *A. tumefaciens*. Left panel: JC2899 (*ΔgcrA* P*tauA-gcrA*) cells were pre-cultured into ATGN + Taurine. Cells were then washed and diluted to an OD_600_∼0.05 into ATGN ± Taurine and placed into a plate reader to record OD_600_ every 15 min. Error bars represent means ± standard deviations from three biologically independent replicates. Right panel: JC2899 cells were pre-cultured in ATGN + Taurine. Once cells reached exponential phase, they were then washed and resuspended to an OD_600_∼0.2 into ATGN ± Taurine. RNA samples were prepared after 3h of growth for qRT-PCR analyses. The graph shows relative mRNA levels in cells cultivated without the Taurine inducer, compared to cells cultivated with the Taurine inducer (then set to an arbitrary value of 1 for each gene = orange dotted line). Three biological replicates with three technical replicates were used for each condition. Significant differences (Wilcoxon rank sum test) when comparing -Taurine/+Taurine are indicated by * (*P*< 0.05) and ** (*P*< 0.01). (**D**) Impact of the three GANTC motifs found in the P_E_ (WT) promoter on plasmid replication/maintenance in *S. meliloti* cells (left) and on promoter activity in *C. crescentus* cells (right). For the P_E_(M1), P_E_(M2) and P_E_(M3) derivatives of P_E_(WT), single methylatable GANTC motifs were mutated into non-methylatable GTNTC motifs. For (B) and (D), minimum 3 independent samples (dots) were used for each construct. Conjugation efficiencies and promoter activities were normalized so that the mean value of the WT construct equals one in each case. Mean values were plotted with error bars corresponding to standard deviations. Student's *t*-tests comparing mutant constructs with WT constructs: ns = not significant (*P*-value > 0.05), ** = *P*-value < 0.001.(**E**) Model for the cell cycle-dependent regulation of the *repABC^Ch2^*/*ori2* module by GcrA. Dashed arrows or ‘?’ indicate still hypothetical regulatory pathways. Wavy lines indicate when transcription from P_A_ (promoter region upstream of the *repABC^Ch2^*operon) or P_E_ (promoter driving RepE^Ch2^ expression) is expected to take place based on their methylation states. When the TSS could not be predicted (for P_A_ and P_G_), it was arbitrarily positioned at the beginning of the ORF.

To test if this over-initiation at *ori2* may be linked with a transient over-expression of the *repABC^Ch2^* operon in these cells, we next compared the *repA^Ch2^/repB^Ch2^/repC^Ch2^* mRNA levels by qRT-PCR analyses on RNA samples extracted from WT cells carrying pRX (the *repABC^Ch2^/ori2* module is then transiently hemi-methylated during the second half of the S-phase of the cell cycle as shown in Figure 1A) or pRX-P*tac-ccrM* (the *repABC^Ch2^/ori2* module is then probably fully-methylated throughout the whole cell cycle) and cultivated in the same ATGN + IPTG conditions. We found that the expression of these three genes was not significantly affected in such a population of *A. tumefaciens* cells with a hyper-methylated genome, compared to control cells (Supplementary Figure S14). This result was not particularly surprising since *ccrM* overexpression is expected to change the methylation state of the *repABC^Ch2^* operon during an only very limited period of the cell cycle of each cell in the population (only during the second part of the S-phase of the cell cycle, estimated as less than 20% of the complete cell cycle). Then, the observed over-initiation at *ori2* when *ccrM* is over-expressed cannot be attributed to a simple excess of *repC^Ch2^* expression. Instead, we believe that preventing the transient hemi-methylation of the GANTC motifs found in the predicted *ori2* (Figure 6A) in these cells may disturb the frequency of replication initiation at *ori2* during the S-phase of the cell cycle as initiation events can easily take place within minutes if conditions are favorable.

### Dissecting the impact of GANTC motifs and GcrA on the expression and the function of the *repABC^Ch2^/ori2* module

Looking more carefully at the *repABC^Ch2^/ori2* module (Figure 6A), we notably found that it carries three GANTC motifs in the promoter region controlling the transcription of the *repABC^Ch2^* operon (P_A_), three GANTC motifs in the intergenic (IG) region located between *repB^Ch2^* and *repC^Ch2^*, and three last ones overlapping the putative *ori2* region inside *repC^Ch2^*. We thus hypothesized that the methylation of some of these motifs by CcrM may affect the expression and/or the function of the different elements of the *repABC^Ch2^/ori2* module.

As a first step to get mechanistic insight into the reasons why CcrM can promote *ori2* replication/partitioning, we cloned the entire *repABC^Ch2^/ori2* module into a vector (pSW25T) that carries a unique conditional R6K origin that can only function in *pir+* bacterial cells (Supplementary Figure S15a). We then tried to move this recombinant plasmid from *pir+ E. coli* donor cells (MFDpir) to *pir- E. coli* recipient cells (DH5α) by conjugation. Interestingly, *trans*-conjugants were not obtained (*trans*-conjugants/recipient cells < 10^−7^), showing that the *repABC^Ch2^/ori2* module is not sufficient for the autonomous replication/maintenance of this recombinant RepABC^Ch2^-dependent plasmid in *pir- E. coli* cells. This observation supports the notion that specific regulators present in *Alphaproteobacteria* but not *Gammaproteobacteria*, such as CcrM and/or GcrA, may play an essential role in allowing *ori2*-dependent replication. We next tried to transfer this RepABC^Ch2^-dependent plasmid into *Sinorhizobium meliloti* cells that do have CcrM and GcrA homologs (note here that we avoided making these tests in *A. tumefaciens* cells as we felt that it would most likely interfere with the replication/partitioning of the *ori2*-dependent chromosome that is essential for cell viability) (Supplementary Figure S15b). Using *S. meliloti* as recipient cells, we obtained many *trans*-conjugants with the pSW25T-repABC^Ch2^(WT) plasmid compared to essentially none with the empty pSW25T’ control (Supplementary Figure S15c), demonstrating that the RepABC^Ch2^/*ori2* module of *A. tumefaciens* contains a functional origin and that it is encoding or finding all the necessary proteins for autonomous replication/partitioning once it enters *S. meliloti* cells by conjugation. We next tested whether any of the three methylatable A bases present in the P_A_ promoter region controlling *repABC^Ch2^*transcription may be required for RepABC^Ch2^-dependent replication. Indeed, site-directed mutagenesis of each of these three methylatable A into non-methylatable T bases (GANTC motifs mutated into GTNTC mutations), showed that two of these A (in M2 and M3 motifs) were critical for RepABC^Ch2^-dependent replication, as *trans*-conjugants were now only very rarely obtained (Figure 6B, left panel). Transcriptional fusions between the WT or the mutated P_A_ promoters (479 bp region upstream of *repA*) and an *egfp* reporter further showed that the A present in the methylatable M3 GANTC motif was critical for transcription from P_A_ in *Caulobacter crescentus* cells (also naturally expressing *ccrM* and *gcrA* homologs) (Figure 6B, right panel). Altogether, these results indicate that M1 has no significant impact on *repABC^Ch2^/ori2* expression or function, while M2 and M3 do play an important role in the ability of *repABC^Ch2^/ori2* to replicate/maintain the recombinant plasmid. The A present in the M3 motif is apparently required for *repABC^Ch2^*transcription and plasmid replication, while the A present in the M2 motif may instead be required for *repABC/ori2-*dependent plasmid partitioning since it is located closer to the two predicted *parS* sites (9) (Figure 6A).

Knowing that GcrA is usually the methylation-sensitive transcriptional regulator working together with CcrM in *Alphaproteobacteria* (15,18,21,47), we next hypothesized that GcrA may also be part of this potential epigenetic mechanism controlling *repABC^Ch2^* expression in *A. tumefaciens*. To test this possibility, we constructed a conditional *gcrA* mutant where the only copy of *gcrA* is under the control of the taurine-inducible P*tauA* promoter. This mutant grew relatively well into ATGN minimal medium complemented or not with the taurine inducer (Figure 6C, left panel), showing that GcrA is not essential for the survival of *A. tumefaciens* in these tested conditions. We then used these same growth conditions to test if GcrA affects *repA^Ch2^, repB^Ch2^* and/or *repC^Ch2^* expression by qRT-PCR (Figure 6C, right panel). As expected, mutant cells cultivated for 3 h without the taurine inducer expressed negligible amounts of *gcrA* compared to cells cultivated with the inducer. This analysis then showed that GcrA has a limited impact on *repA^Ch2^* expression, while it apparently acts as a very significant (direct or indirect) inducer of *repB^Ch2^* and *repC^Ch2^* expression (Figure 6C, right panel).

Since the impact of GcrA on *repC^Ch2^* was much stronger than on *repA^Ch2^*, we hypothesized that the main role of GcrA during *repABC^Ch2^* regulation may be to repress a putative RepE^Ch2^ anti-sense RNA transcribed from the IG region located between *repB^Ch2^* and *repC^Ch2^*, rather than to activate the P_A_ promoter. A closer look at this IG region (Supplementary Figure S16) revealed a striking resemblance with the known P_E_ core promoter of pTi driving the synthesis of the RepE^pTi^ anti-sense RNA that represses *repC^pTi^*transcription and translation (10). To verify that this IG region in *repABC^Ch2^* also includes an active antisense P_E_ promoter, we constructed a transcriptional reporter with *egfp* under the control of this putative P_E_ promoter (96 bp region upstream of the predicted TSS of *repE^Ch2^*) and introduced it into *C. crescentus* cells. Fluorescence measurements showed that this region does contain an active P_E_ promoter (Figure 6D, right panel), confirming the existence of a RepE^Ch2^ antisense RNA that is then expected to repress *repC^Ch2^* expression (Figure 6E). Then, it is likely that GcrA represses this P_E_ promoter to promote *repC^Ch2^* (and *repB^Ch2^*) expression. Since GcrA usually binds to methylated promoters, some of the GANTC motifs found on this P_E_ promoter may have a negative impact on *repE^Ch2^* expression. To test this assumption, we mutated each of the three GANTC motifs found in the P_E_ promoter into non-methylatable GTNTC motifs and tested the impact of these mutations on P_E_ activity in *C. crescentus* cells. This assay showed a limited, but still significant, increase in P_E_ activity when the M1 and M2 motifs of the P_E_ promoter were mutated, while the mutation introduced into the M3 motif apparently blocked most of the activity the promoter (Figure 6D, right panel). This last observation was not particularly surprising since the M3 motif overlaps the predicted -10 region of the P_E_ core promoter (Supplementary Figure S16). This M3 mutation also improved the replication/maintenance of our engineered RepABC^Ch2^-dependent plasmids in *S. meliloti* (Figure 6D, left panel), consistent with the negative impact of RepE^Ch2^ on RepABC^Ch2^-dependent replication. Intriguingly, we observed that the M1 and M2 mutations improved the replication/maintenance of our engineered RepABC^Ch2^-dependent plasmids in *S. meliloti* (Figure 6D, left panel) even if these mutations did not repress P_E_ activity (Figure 6D, right panel). These mutations may, instead, promote the stability or the translation of the polycistronic *repABC^Ch2^* mRNA.

### Dissecting the impact of DNA methylation by CcrM on *gcrA* expression

Knowing that GcrA promotes *repC^Ch2^* expression (Figure 6C), we wished to gather more information on how CcrM-dependent methylation may repress *gcrA* expression (Figure 3). Strikingly, we observed that the 250bp region located upstream of the *gcrA* ORF, which is expected to include the *gcrA* promoter (P_G_), carries 6 GANTC motifs (Supplementary Figure S17a). This is a strong over-representation compared to the average distribution of GANTC motifs on the *A. tumefaciens* chromosome (Supplementary Figure S7). To confirm that P_G_ is in this region and to test whether the methylation of these motifs may repress the activity of P_G_ (consistent with our previous observation that *gcrA* is activated in CcrM-depleted cells shown in Figure 3), we cloned it into the p*lacZ290* vector to create a P_G_-*lacZ* transcriptional reporter. This reporter was then introduced into WT and JC2307 *A. tumefaciens* cells or into *E. coli* cells that expressed or not a catalytically active CcrM MTase to compare the activity of P_G_ by β-galactosidase assays. These assays showed that P_G_ was significantly less active in WT or CcrM-repleted than in CcrM-depleted *A. tumefaciens* cells (Supplementary Figure S17b) and in *Escherichia coli* cells expressing an active CcrM MTase than in control cells expressing a catalytically inactive variant (Supplementary Figure S17c). These results suggest that the *gcrA* promoter is less active when it is methylated than when it is hypo- or non-methylated, even in the absence of Alphaproteobacterial-specific transcriptional regulators since the effect is already detectable in engineered *E. coli* cells that methylate GANTC motifs constitutively. It is then possible that the full methylation of this P_G_ promoter in G1 phase *A. tumefaciens* cells may limit the recruitment of the Sigma factor (or of another conserved transcriptional activator) required for the initiation of *gcrA* transcription. Consistent with this proposition, we also found that *gcrA* mRNA levels were significantly lower in *A. tumefaciens* cells that over-expressed *ccrM* from pRX-P*tac-ccrM* than in control cells carrying the empty pRX control vector (Supplementary Figure S14). Thus, preventing the transient hemi-methylation of P_G_ through CcrM overproduction may reduce P_G_ activity in S-phase cells.

## Discussion

In this study, we discovered that the CcrM DNA MTase, which is conserved in all known *Alphaproteobacteria* with multipartite genomes (11), can play a role in controlling the timing of *ori2* replication/partitioning and the frequency at which *ori2* is replicated in *A. tumefaciens* cells. Our observations indicate that *ori2* initiates/segregates with a significant delay during the *A. tumefaciens* cell cycle (Figure 4 and Figures S11 and S12) when its dicentric chromosome is hypo-methylated due to a depletion of CcrM (Figure 1B and Figures S6 and 7). This replication delay could be due to insufficient levels of the RepC^Ch2^ initiator since *repC^Ch2^* expression is strongly reduced in such mutant cells (Figure 3). Conversely, *ori2* appears to over-initiate when CcrM is over-expressed, leading to cells with unusually high *ori2* copy numbers (Figure 5). We believe that these over-initiation events may result from the constitutive full-methylation of the three GANTC motifs found in the putative *ori2* (Figure 6E), rather than from a very transient mis-regulation of the *repABC^Ch2^* operon during the second half of the S-phase, since we could not detect a noticeable boost in *repABC^Ch2^* expression from mixed cell populations under such conditions (Supplementary Figure S14). In this case, the hemi-methylation of the *ori2* after the onset of replication from *ori2* may be involved in preventing new *ori2* firing before the very end of the S-phase of the cell cycle when CcrM re-methylates the whole genome (model shown in Figure 6E). This would be somewhat reminiscent of the control of DnaA-dependent origins by the Dam/SeqA couple in *Gammaproteobacteria*, where the replication origin is sequestered in an poorly active hemi-methylated state after the initiation of replication to reduce risks of over-initiation events (48). Altogether, we therefore envision that the cell cycle-dependent methylation of the *A. tumefaciens* genome may play a dual role in controlling initiation at *ori2*: first, a role in regulating the timing of the first initiation event at *ori2* through *repC^Ch2^* regulation and, second, a role in regulating *ori2* initiation frequency through the transient hemi-methylation of the *ori2* after initiation. This impact of CcrM-dependent methylation on *ori2* control may also provide an explanation for the apparent essentiality of *ccrM* in cells cultivated in complex media (Figure 2 and Supplementary Figure S2) since initiation at *ori2* is essential in *A. tumefaciens* (28). *ori2* and/or P_A_/P_E_ methylation by CcrM may be needed to reset *ori2* activity and/or *repC^Ch2^* expression once cells have reached the G2 phase of the cell cycle and need to get ready to restart a new cell cycle (model in Figure 6E). Why this process becomes more critical in complex media may, for example, relate to higher coordination requirements during fast growth or to shorter half-lives of critical regulators (CcrM, RepABC^Ch2^, …) leading to more critical depletions of such elements. Interestingly, such CcrM-dependent control of secondary chromosomal origins may be conserved far beyond *A. tumefaciens*, as a former study showed that CcrM over-expression leads to abnormally high intra-cellular DNA contents in the *B. abortus* human pathogen (22).

How DNA methylation by CcrM promotes initiation from *ori2* in *A. tumefaciens* is however not yet fully understood. Still, our data indicates that the A present in several GANTC motifs located in the P_A_ promoter controlling the transcription of the *repABC^Ch2^* operon (M2 and M3 in P_A_) and in the P_E_ promoter controlling the synthesis of the RepE^Ch2^ anti-sense RNA (M1, M2 and M3 in P_E_), can decrease or increase, respectively, the activity of *ori2 in cis* on a plasmid (Figure 6b,d). While a few of these methylatable A may simply be part of the core promoters required for Sigma factor binding (M3 motifs in P_A_ and P_E_), others have a more minor impact on promoter activity while they still influence *ori2* activity (M2 in P_A_ and M1/M2 in P_E_). These may be regulatory GANTC motifs influencing *repE^Ch2^*transcription (M1/M2 in P_E_) and/or other events required for *ori2* activity/partitioning (M1/M2 in P_E_ and P_A_). Considering that the three genes of the *repABC^Ch2^*operon are down-regulated when the *A. tumefaciens* genome becomes hypo-methylated (Figure 3A, B), we envision that methylation of the A in the M3 motif of the P_A_ promoter by CcrM may promote *repABC^Ch2^* transcription (model in Figure 6E). If this is the case, the GcrA epigenetic regulator would not play a major role, as its depletion had an only minor impact on *repA^Ch2^* expression in *A. tumefaciens* (Figure 6C). In contrast, GcrA showed a strong positive impact of *repC^Ch2^* expression, suggesting that it can repress the RepE^Ch2^ sRNA (model in Figure 6E). This newly detected antisense RNA (Figure 6D) shows striking resemblances with the RepE^pTi^ sRNA (Supplementary Figure S16) that has been shown to repress not only *repC^pTi^* transcription (through premature transcriptional termination of the *repABC^pTi^*multicistronic transcript upstream of the *repC^pTi^* ORF), but most likely also *repC^pTi^*translation (10). Since we observed that RepABC^Ch2^/*ori2*-dependent plasmids carrying GANTC mutations repressing *repE^Ch2^* expression enter and are maintained more efficiently into cells than plasmids carrying the WT module (Figure 6D), we believe that RepE^Ch2^ is similarly a potent inhibitor of *repC^Ch2^* expression (model in Figure 6E). Considering that GcrA is usually a methylation-sensitive regulator, its impact on P_E_ may be modulated by the methylation state of its three GANTC motifs. Altogether, it is then possible that DNA methylation by CcrM has a dual impact on *repC^Ch2^* expression, by promoting its transcription from the P_A_ promoter (M3 motif) and through an inhibition of the P_E_ promoter *via* the GcrA epigenetic regulator (model in Figure 1E).

Another important question is why should this RepABC^Ch2^/*ori2* module be regulated by such complex epigenetic mechanisms of regulation? An attractive answer may be that such a system could be used as a sophisticated timer to activate *ori2* only once the DnaA-dependent *ori1* has already started the replication of the chromosome as observed (Figure 4). This would then play a role in coordinating the initiation of replication at the two chromosomal origins, which is important for genome maintenance. Indeed, our data suggests that the *gcrA* promoter (P_G_), which is located at a distance of ∼0.43 Mbp from the *ori1* (Figure 1B), may be activated when it switches from a fully- to a hemi-methylated state once the replication fork reaches that locus following initiation at *ori1*. A burst of GcrA synthesis at that early time of the S-phase of the cell cycle may then repress the fully-methylated RepE^Ch2^ anti-sense RNA, leading to a strong activation of *repC^Ch2^* expression (model in Figure 6E). RepC^Ch2^ would then accumulate at sufficiently high levels to start replication at *ori2* significantly after *ori1*, but in a coordinated manner. Once replication has started at *ori2*, over-initiation from *ori2* may then be prevented through the hemi-methylation of *ori2* (model in Figure 6E), as seen for DnaA-dependent origins in some *Gammaproteobacteria* (48). To complete this working model, future studies should notably focus on testing the impact of DNA methylation on the cell cycle-regulation of GcrA levels and on testing whether GcrA and/or RepC are methylation-sensitive DNA binding proteins in *A. tumefaciens*. Such studies can shed interesting light on the complex (epigenetic) regulatory networks that primitive organisms with complex genomes can use to coordinate genome replication from multiple origins the way more complex organisms also do.

Beyond the observed impact of CcrM on *repABC^Ch2^* expression, our transcriptome analysis revealed that DNA methylation by CcrM modulates the expression of many other *A. tumefaciens* genes. Gene Set Enrichment Analysis (GSEA) tests showed enrichments for COG categories J (translation/biogenesis), L (replication/recombination/repair) and N (cell motility) when comparing the transcriptome of CcrM-depleted cells with that of WT or CcrM-repleted cells (Supplementary Figure S18). The COG category N correlates with the motility/adhesion defects observed for CcrM-depleted cells (Supplementary Figure S8), while the COG category L may correlate with up-coming replication issues, even if initiation from *ori2* still appeared as normal in most CcrM-depleted cells after 7h of growth in ATGN - IPTG (data not shown). Among the genes that were significantly mis-regulated upon CcrM depletion, up to 75 may be directly regulated by CcrM-dependent methylation (Figure 3A and Supplementary Table S5) and this ‘direct regulon’ is apparently very different from previously described CcrM regulons in distantly-related bacteria (11,21) (Supplementary Table S5). Moreover, the essential *ftsZ^At^* gene of *A. tumefaciens* (that has one GANTC motif in the 200 bp upstream of the ORF) was not significantly down-regulated in CcrM-depleted cells cultivated in complex media (Figure 3B), contrarily to what was previously observed in *C. crescentus* *ΔccrM* mutant cells where FtsZ^Cc^ levels can become too limiting to sustain cell division and viability (19,20). We then propose that the essentiality of *ccrM* in *A. tumefaciens* is instead connected with its impact on *repC^Ch2^* expression and/or *ori2* methylation/activity to ensure genome maintenance since *ori2* is apparently essential in *A. tumefaciens* (replication forks may stall at the *ter1* region located between *ori1* and *ori2*) (28). The elongated cell phenotype observed for CcrM-depleted cells cultivated in complex media before they die (Figure 2C and Supplementary Figure S2 and S4) may also be connected with this replication defect as cell division is often delayed when chromosome replication/segregation is stuck in bacteria. Overall, even if *ccrM* is often (conditionally) essential in *Alphaproteobacteria* (19,22,23,49), the reasons why *ccrM* can become essential appears to vary from one species to another, confirming the interest of studying the biological function of conserved DNA MTases in a variety of organisms even if their molecular function appears as identical.

## Supplementary Material

gkae757_Supplemental_Files

## Data Availability

Metadata and RNASeq/SMRT-Seq data are available in the NCBI BioProject PRJNA1064157: https://www.ncbi.nlm.nih.gov/search/all/?term=PRJNA1064157. Other data that support the findings of this study are available from the corresponding author upon request.
