# Supplementary material for: DNA methylation by CcrM contributes to genome maintenance in the Agrobacterium tumefaciens plant pathogen
Source: Nucleic Acids Res. 2024 Sep 4;52(19):11519–35. doi: 10.1093/nar/gkae757 (PMC11514494; doi:10.1093/nar/gkae757)
Supplement: gkae757_Supplemental_Files [file gkae757_supplemental_files.zip › SupInfo_Revised_Collier.pdf]

SUPPLEMENTARY INFO

DNA methylation by CcrM contributes to genome maintenance in the *Agrobacterium tumefaciens* plant pathogen

Sandra MARTIN, Florian FOURNES, Giovanna AMBROSINI, Christian ISELI, Karolina BOJKOWSKA, Julien MARQUIS, Nicolas GUEX and Justine COLLIER

CONTENT:

Page 2: SUPPLEMENTARY FIGURES WITH LEGENDS

Page 16: SUPPLEMENTARY TABLES WITH CAPTIONS

Page 23: SUPPLEMENTARY METHODS

Page 26: SUPPLEMENTARY REFERENCES

SUPPLEMENTARY FIGURES WITH LEGENDS:

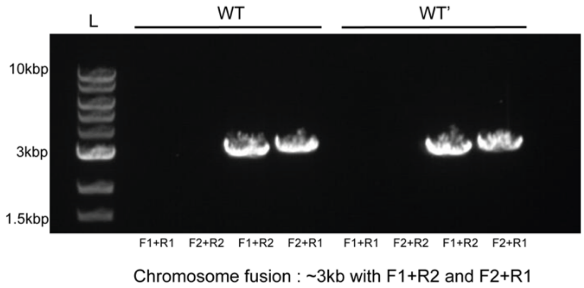

Figure S1: PCR and gel electrophoresis analyses showing that JC2140 (WT) and JC2141 (WT') *A. tumefaciens* C58 cells display a unique dicentric chromosome as described in (1). gDNA from WT and WT' cells were analyzed by PCR using F1/F2/R1/R2 primers as done before (1). Detection of a PCR product with F1/R2 and F2/R1 primer pairs and no PCR product with F1/R1 and F2/R2 primer pairs indicates that JC2140 (WT) and JC2141 (WT') display a unique dicentric chromosome, which was also further confirmed by whole-genome sequencing and assembly. L: DNA ladder.

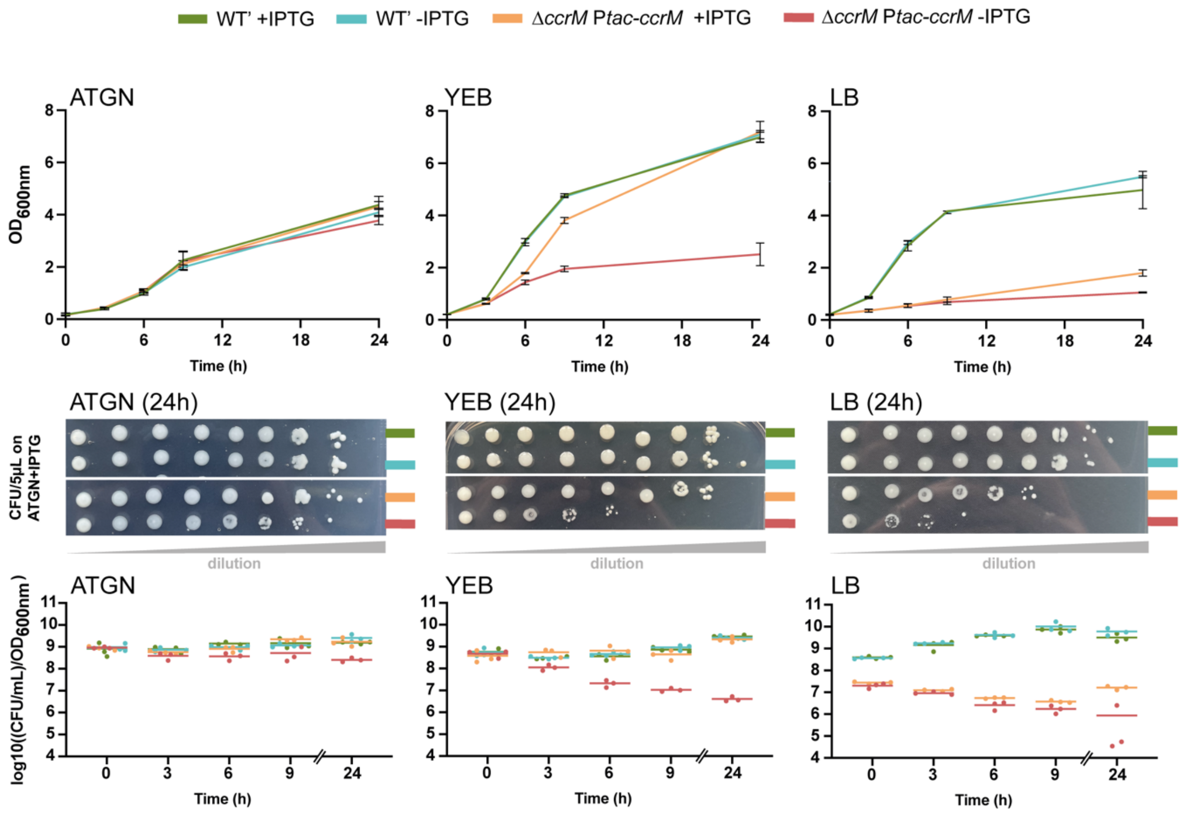

Figure S2: CcrM-depleted cells loose viability over time when cultivated in complex media. (a) JC2141 (WT') and JC2307 (*ΔccrM* P*tac-ccrM*) cells were pre-cultivated into ATGN+IPTG medium and cultures were diluted back into the indicated media +/-IPTG in test tubes. Growth was evaluated over-time measuring the OD_{600}. **(b)** After 24h of growth from (a), 5µL samples of each culture were collected and spotted onto solid ATGN+IPTG medium (= first undiluted sample on the left of each plate lane). Serial 10-fold dilutions of each culture were also plated (from left to right sides of images) to compare the capacities of cells to form colony forming units (CFU) on permissive plates (JC2307

grows relatively well onto ATGN+IPTG medium as can be seen in Fig.2) depending on the liquid medium into which cultures were grown. **(c)** Quantitative comparison of CFU using three biological replicates for each condition/strain (one example of each is shown in panel (b)).

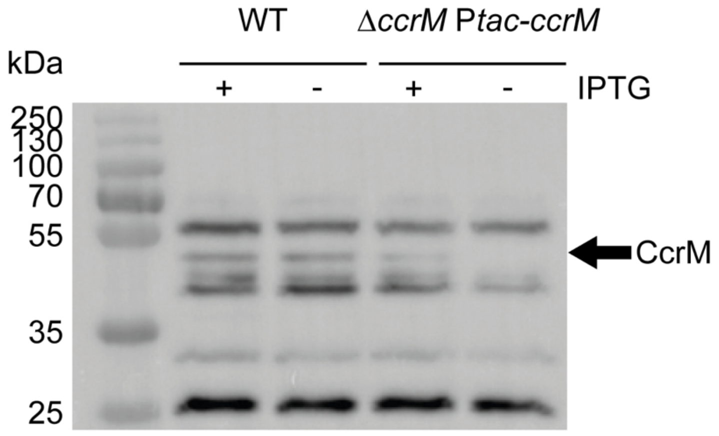

Figure S3: Immunoblot experiments showing that CcrM is depleted in JC2307 cells cultivated in ATGN-IPTG for 7 hours. JC2140 (WT) and JC2307 (*ΔccrM* P*tac-ccrM*) cells were cultivated into ATGN+/-IPTG as described in Fig.S2a and samples were collected at the 7-hour time point. Proteins were separated on a 12% SDS-PAGE gel and transferred onto a PVDF membrane (Millipore). Immunoblotting was performed following a standard protocol (2) and using a rabbit anti-CcrM^{At} serum (generous gift from the Viollier lab, University of Geneva) diluted 1:5000 and a goat anti-rabbit (A9169 from Sigma) diluted 1:30000. The black arrow indicates which protein most likely corresponds to CcrM (depleted when IPTG is removed and fitting the expected molecular weight of CcrM^{At} of ~42.2 kDa). Other detected proteins can be used as loading controls.

→ Also note that the exact same ATGN+/-IPTG culture samples as described here were used for the SMRT-Seq (Fig.1b and Fig.S7 below), RNA-Seq (Fig.3 and Fig.S9 below) and HinfI-based (Fig.S6 below) experiments described in this study.

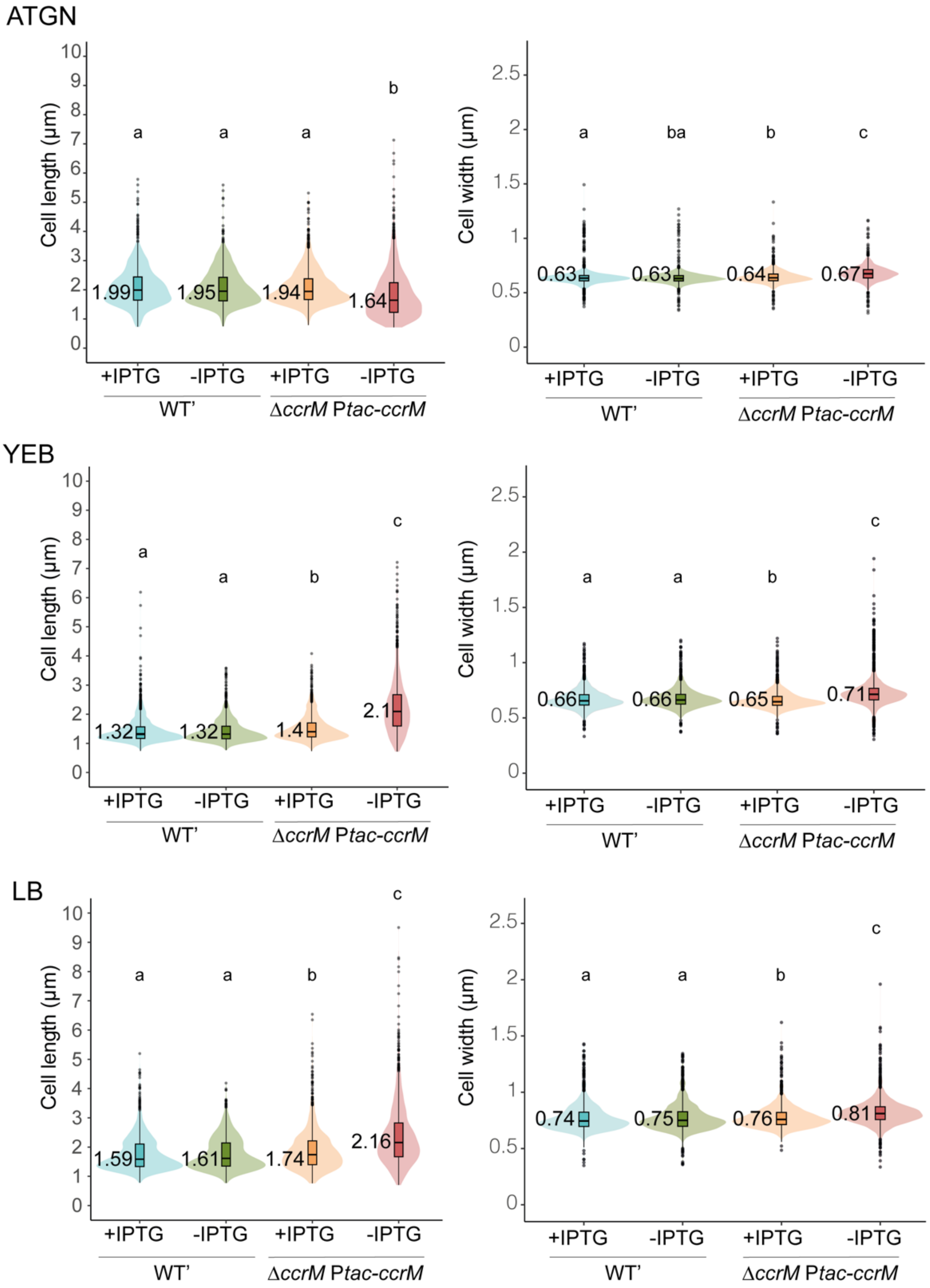

Figure S4: Microscopy image analyses comparing the length/width of JC2141 (WT') and JC2307 (*ΔccrM* P*tac-ccrM*) cells cultivated in different media. Strains were cultured and imaged as described in Fig.2c (24 hours of growth in the indicated media). The length and the width of 1500 cells for each strain/condition were then measured from phase-contrast images using the Fiji 2.3.0 software with the MicrobeJ plugin (3). Written values indicate the median cell length or width in the cell population. Boxplots include the 25-75 percentiles. The potential significance of differences was evaluated for each plot using Kruskal-Wallis tests with Bonferroni corrections. Note that differences in cell widths are globally subtle even if 2-3 statistically different groups (a, b or c) can be distinguished (P-value<0.001) for each plot using this test.

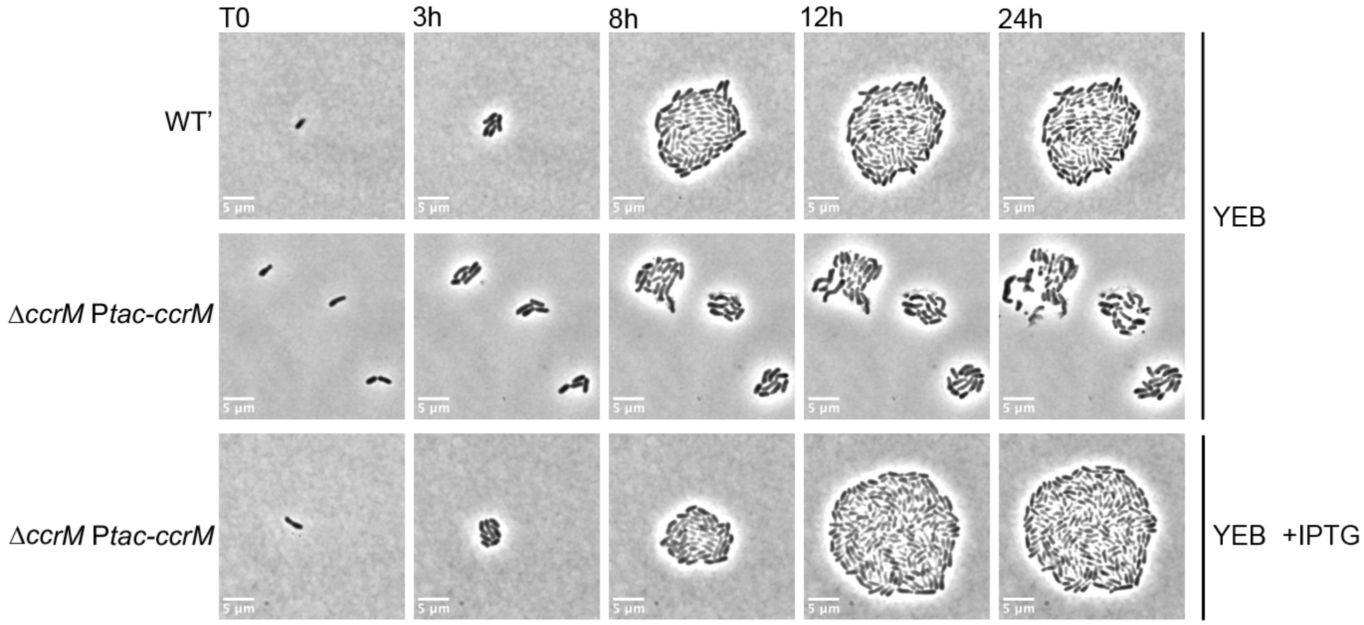

Figure S5: Time-lapse microscopy images of JC2141 (WT') and JC2307 *(ΔccrM* P*tac-ccrM*) cells cultivated in YEB +/-IPTG. Strains were pre-cultured to stationary phase in YEB+IPTG and then diluted back into YEB+IPTG. Once cells reached exponential phase again, they were washed and resuspended into YEB+/-IPTG to be spotted onto a YEB+/-IPTG agarose (1%) pads (T0) for phase-contrast microscopy. Cells were imaged every 15 minutes for 24 hours using a 100x/1.40 inverted oil-immersion objective on a Leica DMi8 microscope with a sCMOS DFC9000 (Leica) camera and SOLA light engine (Lumencor). Representative images of developing micro-colonies are shown in this figure. Movies showing microcolony growth over time for these three strains/conditions are also available: Movie S1 (JC2141 on YEB -IPTG), Movie S2 (JC2307 on YEB -IPTG) and Movie S3 (JC2307 on YEB +IPTG).

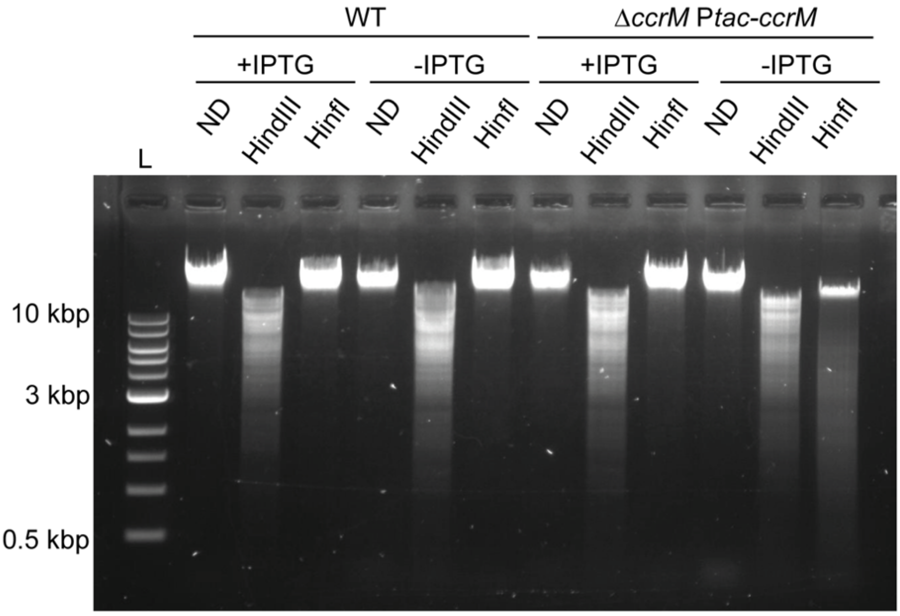

Figure S6: HinfI-based assay indicating that the genome of CcrM-depleted cells becomes hypo-methylated. JC2140 (WT) and JC2307 (*ΔccrM* P*tac-ccrM*) cells were cultivated into ATGN+/-IPTG as described in Fig.S2a and samples were collected at the 7-hour time point for gDNA extraction. 2 µg of gDNA was kept non-digested (ND) or digested with HindIII (digestion control, independent of DNA methylation state) or HinfI (cuts only at non-methylated GANTC motifs) for 4 hours at 37˚C. gDNA fragments were then separated by gel electrophoresis using a TAE 1X with 0.8% agarose gel. L: DNA ladder.

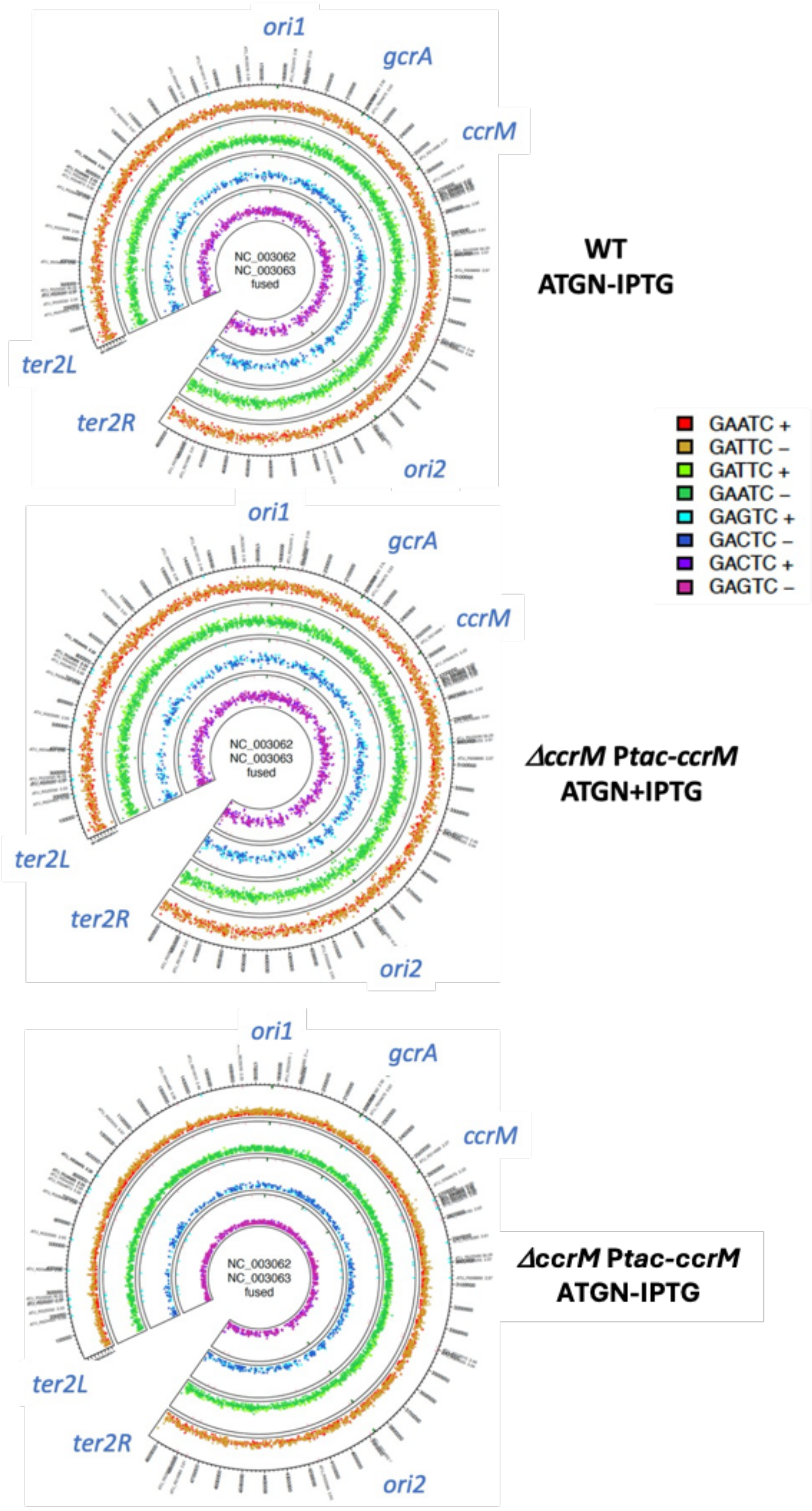
ori1
gcrA
ccrM
ter2L
ter2R
ori2
NC_003062
NC_003063
fused
WT
ATGN-IPTG
ΔccrM Ptac-ccrM
ATGN+IPTG
ΔccrM Ptac-ccrM
ATGN-IPTG
GAATC +
GATTC –
GATTC +
GAATC –
GAGTC +
GACTC –
GACTC +
GAGTC –

Figure S7 (previous page): **Distribution and IPD ratio (y axis) of individual GANTC motifs (+ or – strands) on the dicentric linear chromosome (from *ter2L* to *ter2R*) of JC2140 and JC2307 cells cultivated in ATGN+/-IPTG for 7 hours.** Cells were cultivated as described in Fig.S2a and samples were collected at the 7-hour time point for gDNA extraction followed by SMRT-Seq as also described in Fig.1b. The same y scale (for IPD ratio) was used in all three schematics.

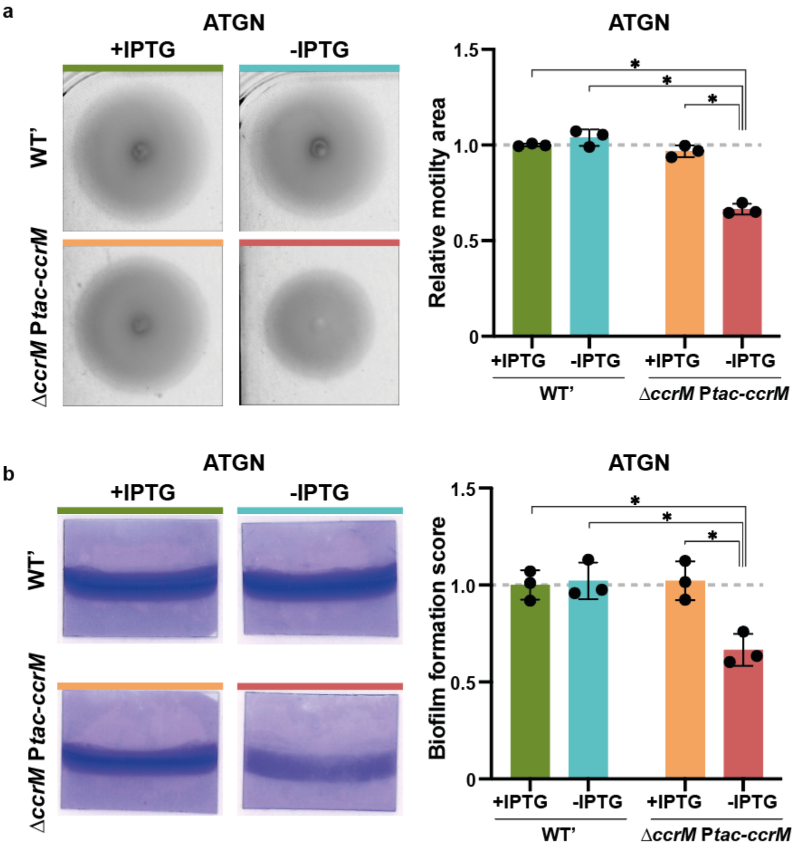

Figure S8: Depletion of CcrM results in motility and biofilm formation defects in minimal medium. (a) CcrM-depleted cells are less motile on soft-agar plates. Swim plate assays were conducted as described previously (4) with minor adaptations. JC2141 (WT') and JC2307 (Δ*ccrM* P*tac-ccrM*) strains were pre-cultured into liquid ATGN+IPTG. Over-night cultures were then washed and adjusted to an OD_{600}~0.6 into ATGN. 5µL were then spotted onto ATGN soft-agar plates +/-IPTG and plates were incubated for 48 hours prior to imaging. Representative images are shown on the left. Quantitative analysis using images from 3 biological replicates with 2 technical replicates each are shown on the right (relative to WT' cells cultivated in ATGN+IPTG). Swimming areas were measured from images using the ImageJ 2.3.0 software. Error bars represent means +/- standard deviations from the three biologically independent replicates (shown as black circles). **(b)** CcrM-depleted cells display attachment/biofilm formation defects. JC2141 and JC2307 strains were pre-cultured into liquid ATGN+IPTG. Over-night cultures were then washed and adjusted to an OD_{600}~0.05 into ATGN+/-IPTG. Cultures were then assayed for biofilm formation on vertical plastic coverslips immersed into the culture. Representative images of coverslips after a 72-hour incubation period at room temperature are shown on the left. Biofilm scores were then measured using data from 3 biological replicates and are shown on the right (relative to WT' cells cultivated in ATGN+IPTG). Error bars represent means +/- standard deviations from the three replicates (shown as black circles). For both panels, a * indicates a significant difference (P<0.01) using an ANOVA (ANalysis Of VAriance) with Tukey HSD (Hosnestly Significant Difference) test.

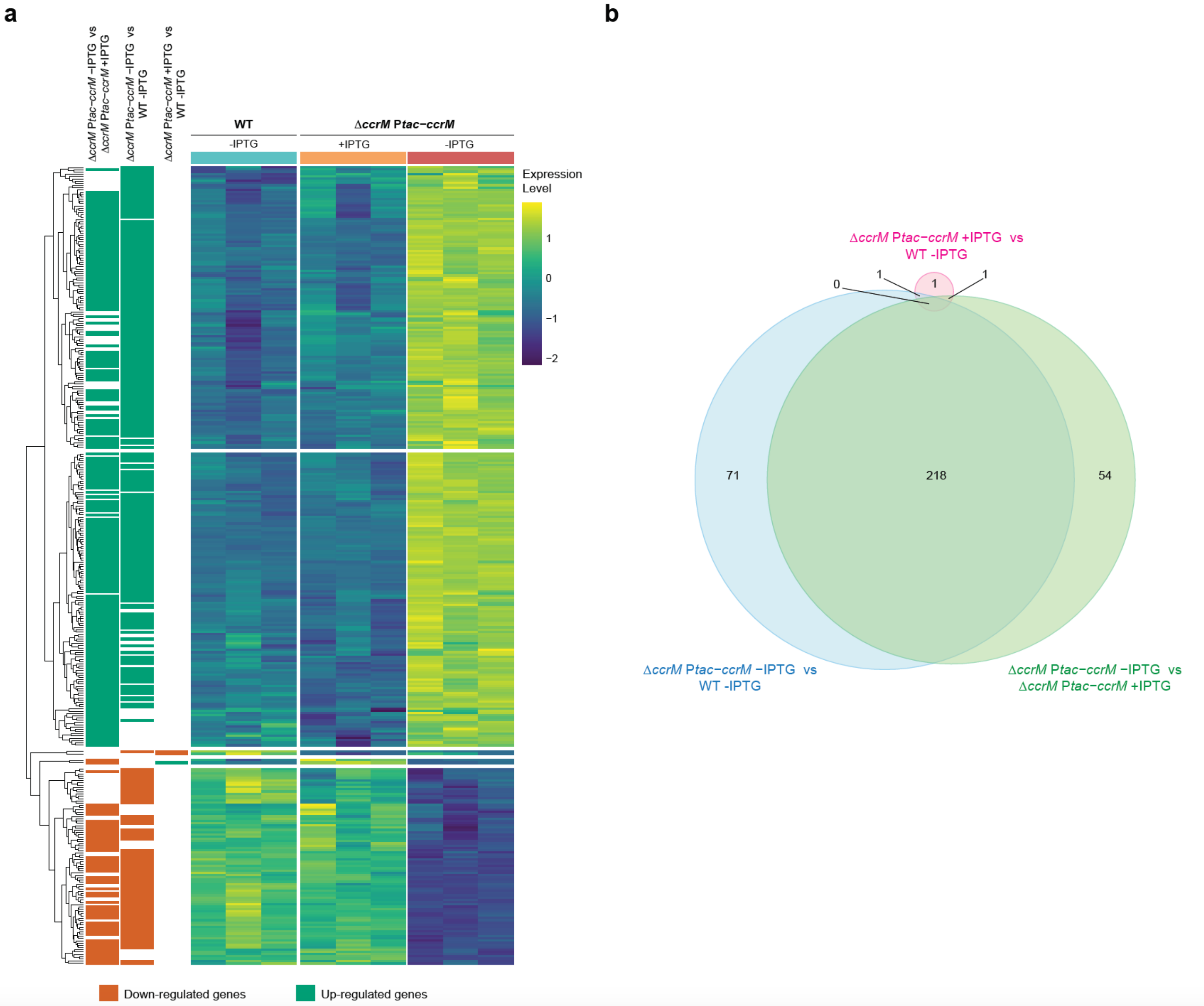

Figure S9: Comparison of the transcriptome of JC2140 and JC2307 cells cultivated in ATGN +/- IPTG for 7 hours. Cells were cultivated into ATGN+/-IPTG as described in Fig.S2a and samples were collected at the 7-hour time point for RNA-seq analyses. **(a)** Heatmap of significantly mis-regulated genes (adj.P-value<0.01 and minimum 2-fold change), ordered by genotypes: WT (JC2140) -IPTG, Δ*ccrM* P*tac-ccrM* (JC2307) +IPTG and Δ*ccrM* P*tac-ccrM* -IPTG. Expression values for each gene were scaled and centered. Colors on the left-hand side indicate if genes are down-regulated (orange) or up-regulated (green). **(b)** Venn diagram showing the number of significantly mis-regulated genes from panel **(a)**. Each circle corresponds to one comparison and the circle size partially reflects the number of mis-regulated genes in the given comparison.

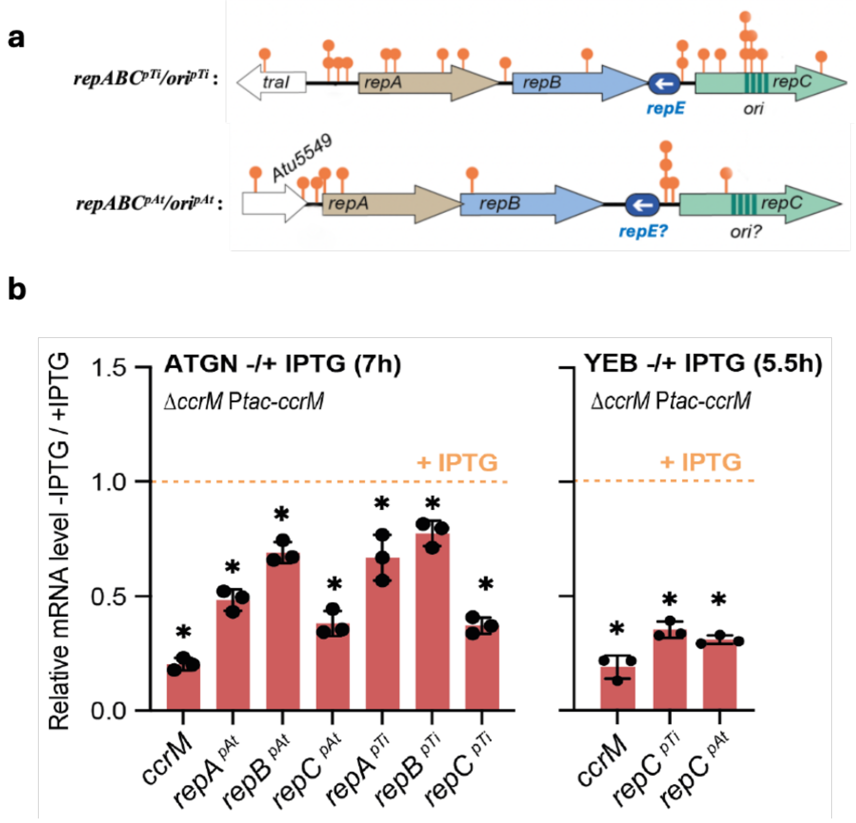

Figure S10: DNA methylation by CcrM promotes the expression of the replication initiators of the two megaplasmids of *A. tumefaciens*. (a) Schematic showing the approximate location of GANTC motifs (orange lollipops) on the *repABC* operons of pTi and pAt. Note that the ORFs are not to scale in this schematic. **(b)** qRT-PCR results confirming the impact of CcrM on the expression of a selection of genes in JC2307 (Δ*ccrM* P*tac-ccrM*) cells cultivated in minimal (left) and complex (right) media. The graphs show relative mRNA levels in cells cultivated without the IPTG inducer, compared to cells cultivated with the IPTG inducer (then set to an arbitrary value of 1 for each gene = orange dotted line). The same RNA samples as those described in Fig.3 were used here. For both panels, three biological replicates with three technical replicates each were used. Significant differences (Wilcoxon rank sum test) when comparing -IPTG/+IPTG are indicated by * (P<0.001).

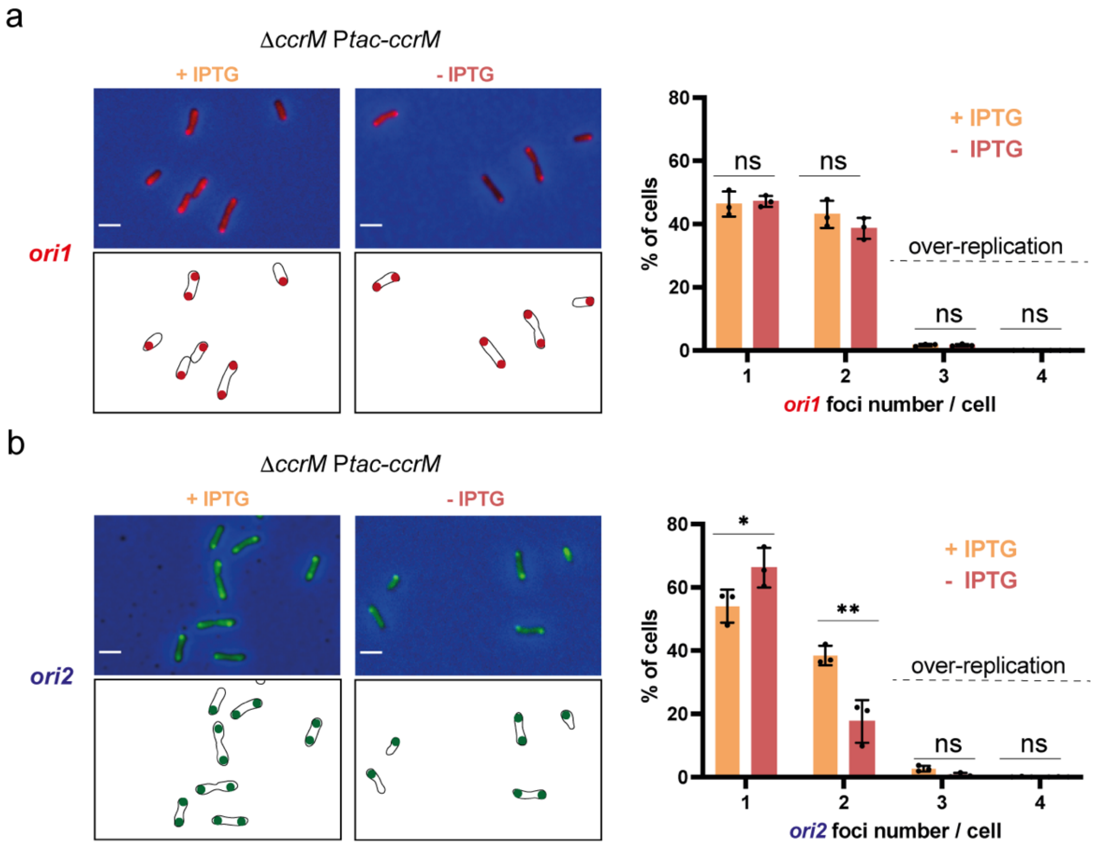

Figure S11: Localization and number of *ori1* or *ori2* (single *ori* labelling) in CcrM-repleted and CcrM-depleted cells cultivated in ATGN medium. Left side: Selected microscopy images of JC2660 (*ΔccrM* P*tac-ccrM* with *ori1/ygfp* reporter) cells in panel **(a)** or JC2661 cells (*ΔccrM* P*tac-ccrM* with *ori2/ygfp* reporter) cells in panel **(b)** cultivated over-night in ATGN+/-IPTG. Cultures were then diluted into ATGN+/-IPTG and grown exponentially for ~6.5 hours (same medium at all steps). Upper panels: overlays of phase contrast and YGFP images. Lower panels: schematics showing *ori1* (red color in **(a)**) or *ori2* (green color in **(b)**) subcellular localization in the cells imaged above. **Right side:** Quantification of *ori1* or *ori2* number per cell from multiple microscopy images (including minimum 1000 cells). Mean values from 3 independent experiments were plotted for each strain/condition. Error bars correspond to standard deviations. Student's t-test: ns: P-value>0.05, *: P-value < 0.05, **: P-value<0.01.

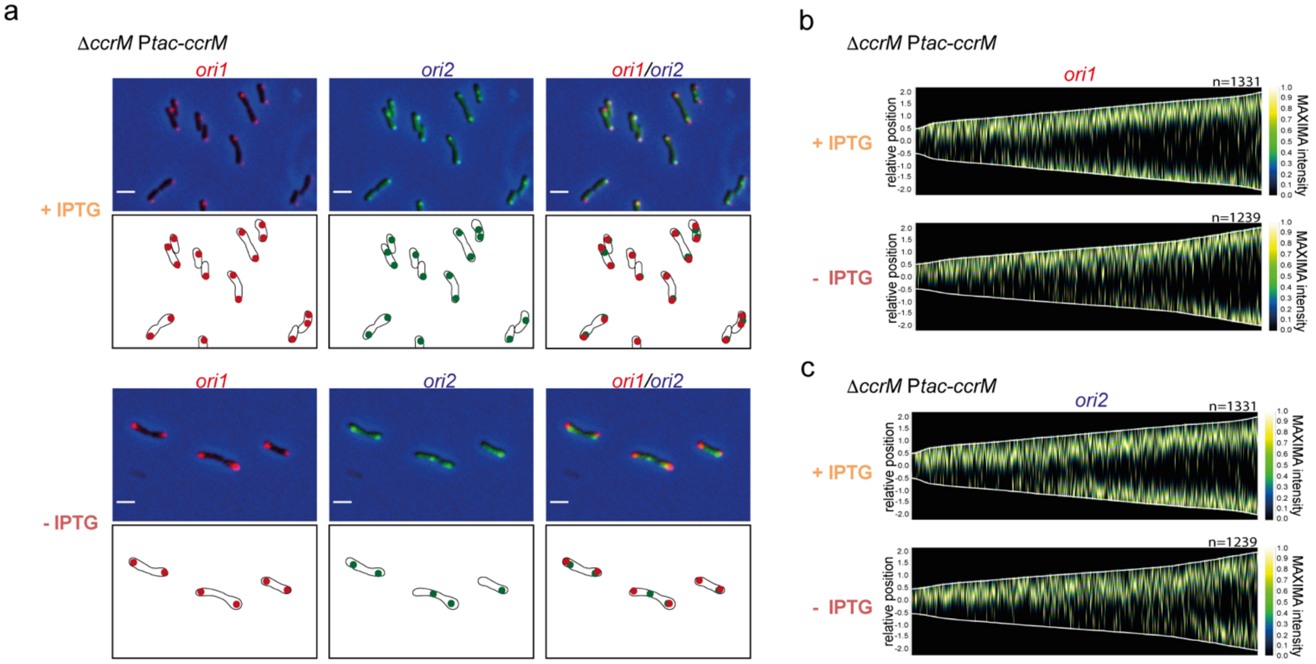

Figure S12: Localization and number of *ori1* and *ori2* (double *ori* labelling) in CcrM-repleted and CcrM-depleted cells cultivated in ATGN medium. (a) Selected microscopy images of JC2836 (*ΔccrM* P*tac-ccrM* with *ori1/mcherry* and *ori2/ygfp* reporters) cells cultivated over-night in ATGN+/-IPTG. Cultures were then diluted into ATGN+/-IPTG and grown exponentially for ~6.5 hours (same medium at all steps). Upper panels: overlays of phase contrast and YGFP and/or mCherry images. Lower panels: schematics showing *ori1* (red color) or *ori2* (green color) subcellular localization in the cells imaged above. **(b and c)** Demographs showing the subcellular localization of *ori1* **(b)** and *ori2* **(c)** foci as a function of cell size (same conditions as in panel **(a)**). Relative position = 0 corresponds to mid-cell. Only cells measuring from 1 to 4 µm-long were included into these demographs. n: number of cells used to construct each demograph.

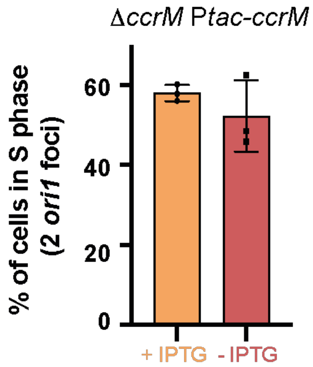

Figure S13: Proportion of S-phase cells in JC2836 (*ΔccrM* P*tac-ccrM* with *ori1/mcherry* and *ori2/ygfp* reporters) cells. Cells were cultivated over-night in ATGN+/-IPTG. Cultures were then diluted into ATGN+/-IPTG and grown exponentially for ~6.5 hours (same medium at all steps) as described in Fig.S9. The number of *ori1* (red foci)/cell was measured as a *proxi* to evaluate the % of S-phase cells in each population. Three independent cultures were used for each condition (minimum 500 cells/condition). Mean % of cells were plotted. Error bars correspond to standard deviations. A student's t-test indicates that the difference is not significant (P-value = 0.19).

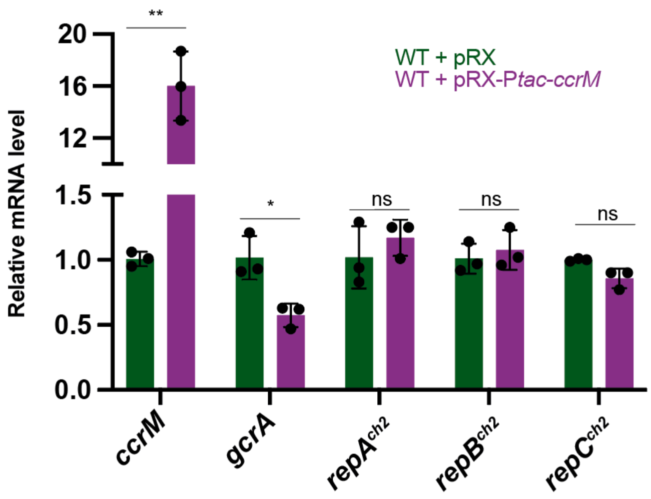

Figure S14: The impact of CcrM over-expression on *repABC*^{Ch2} and *gcrA* expression. RNA samples were prepared from WT cells carrying pRX-P*tac-ccrM* or the empty pRX vector. Cells were cultivated over-night in ATGN+IPTG. Cultures were then diluted into ATGN+IPTG and grown exponentially for ~6.5 hours. Differences in gene expression were evaluated based on the stable *hemF* internal gene control. The graph shows relative mRNA levels in cells carrying the pRX-P*tac-ccrM*, compared to cells carrying pRX (then set to an arbitrary value of 1 for each tested gene). Three independent biological samples (dots) were used for each strain and mean values were plotted with error bars corresponding to standard deviations. Student's t-tests: ns = P-value>0.05, * = P-value<0.05, ** = P-value<0.01.

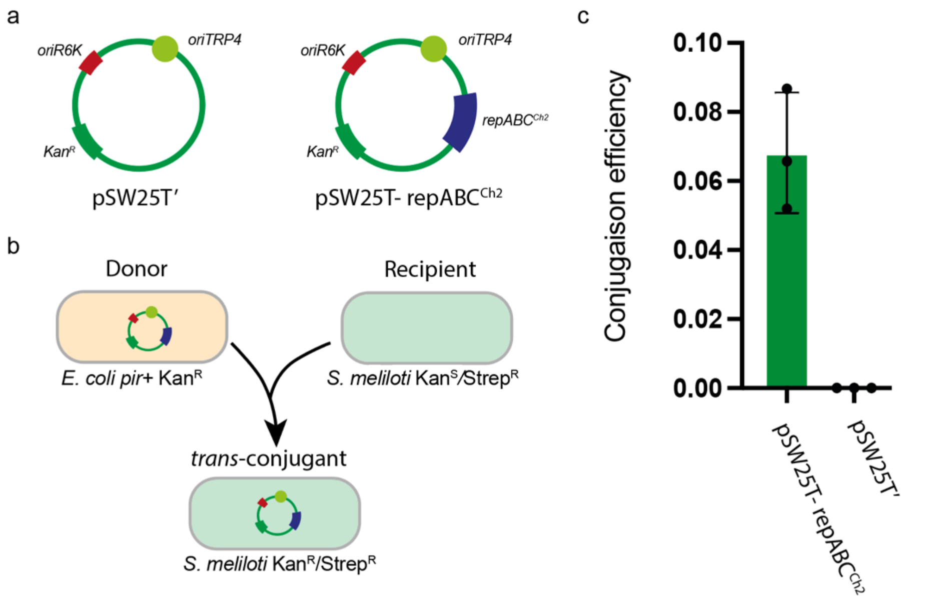

Figure S15: The *repABC*^{Ch2}*/ori2* module is sufficient for plasmid replication/maintenance in *S. meliloti*. (a) Schematic describing the empty pSW25T' vector and its pSW25T-repABC^{Ch2}(WT) derivative. Kan^{R} = kanamycin-resistance gene; *oriR6K*: replication origin that is only functional in *pir+* bacterial cells; *oriTRP4*: origin of transfer. **(b)** Experimental set-up to quantify the efficiency of transfer of vectors from *pir+ E. coli* cells (JC2916) to streptomycin-resistant (Strep^{R}) *S. meliloti* cells (JC2707). **(c)** Measured conjugation efficiency = number of *trans*-conjugants (Strep^{R}/Kan^{R})/number of recipient cells (Strep^{R}/Kan^{R} or Kan^{S}). Three independent conjugation assays (dots) were done using each vector and mean values were plotted with error bars corresponding to standard deviations.

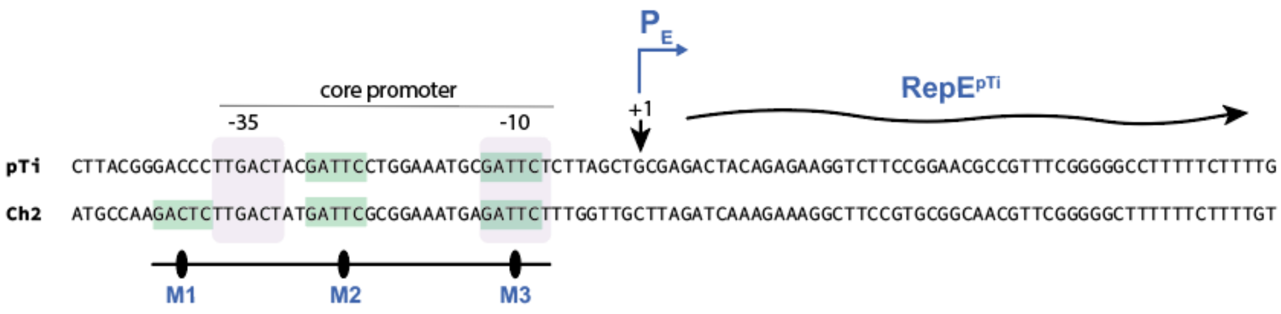

Figure S16: Schematic comparing the known P_E^{pTi} promoter region (5,6) with the predicted P_E^{Ch2} promoter region on the *A. tumefaciens* genome. -10/-35 core promoter elements and the +1 TSS are shown. Methylatable GANTC motifs (named M1/M2/M3 for the P_E^{Ch2}) are highlighted in green. The wavy line shows the length of $RepE^{pTi}$.

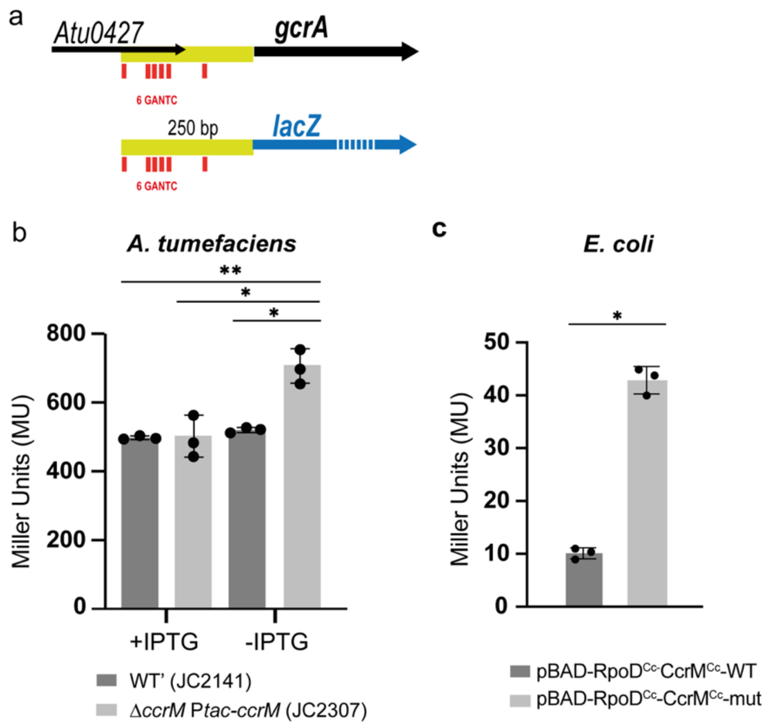

Figure S17: CcrM represses the *A. tumefaciens gcrA* promoter in *A. tumefaciens* and *E. coli* cells. **(a)** Map of the *gcrA (Atu0426)* promoter region (yellow) and position of GANTC motifs (red) in this region on the *A. tumefaciens* genome (upper panel) and schematic of the 250bp region cloned into p*lacZ*290 to create a P*gcrA-lacZ* transcriptional reporter (into p*lacZ*290-P*gcrA* that can replicate into *E. coli* and *A. tumefaciens* cells). *lacZ* was represented with a dashed blue line as it is longer than *gcrA*. **(b)** p*lacZ*290-P*gcrA* was introduced into the indicated *A. tumefaciens* C58 derivatives. Cells were cultivated into ATGN+IPTG until cultures reached an OD_{600}~0.4. Cells were then washed and resuspended into ATGN+/-IPTG at an OD_{600}~0.05. Cultures were then incubated for 14 hours before samples were collected for β-galactosidase assays to evaluate the activity of the *gcrA* promoter in *A. tumefaciens*. Significant differences (ANOVA (ANalysis Of VAriance) with Tukey HSD test) are indicated by * (P<0.01) or ** (P<0.001). **(c)** p*lacZ*290-P*gcrA* was introduced into *E. coli* TOP10 cells carrying pBAD-$RpoD^{Cc}$-$CcrM^{Cc}$-WT (expressing the *C. crescentus* RpoD and CcrM proteins) or pBAD-$RpoD^{Cc}$-$CcrM^{Cc}$-mut (expressing the *C. crescentus* RpoD protein and an inactive variant of its CcrM protein). Cells were cultivated over-night into LB and then resuspended into LB+arabinose 0.3% at an OD_{600}~0.05. Cultures were then incubated for 3 hours before samples were collected for β-galactosidase assays to evaluate the activity of the *gcrA* promoter in *E. coli* cells expressing the *C. crescentus* $RpoD^{Cc}$ housekeeping Sigma factor together with an active or inactive $CcrM^{Cc}$ protein.

Significant difference (Student's t-test) is indicated by * (P<0.0001). For all β-galactosidase assays (panels b and c), three biological replicates were done.

**COG category**	***ΔccrM* P*tac-ccrM* +IPTG versus WT-IPTG**	***ΔccrM* P*tac-ccrM* -IPTG versus WT-IPTG**	***ΔccrM* P*tac-ccrM* -IPTG versus *ΔccrM* P*tac-ccrM* +IPTG**
**J (translation/biogenesis)**	NS	P-value=0.0115	P-value=0.0115
**L (replication/recombination/repair)**	NS	P-value=0.0115	P-value=0.0115
**N (cell motility)**	NS	P-value=0.0153	NS

Figure S18: COG categories J, L and N are significantly over-represented among genes that are mis-regulated in CcrM-depleted cells compared to CcrM-repleted or WT cells. Strains, growth conditions and RNA samples are the same as described in Fig.S9. A Gene Set Enrichment Analysis (GSEA) was performed using the R package BOG (Bacterium and virus analysis of Orthologous Groups) (7) to identify over-represented COG categories among genes that were significantly mis-regulated (FC>2 and adjusted P-value<0.01 in Table S4) when comparing the indicated strains/conditions. P-values were adjusted for multiple testing. NS = no significant enrichment.

SUPPLEMENTARY TABLES:

Table S1: Strains used in this study

**Strain name**	**Genotype**	**Description**	**Reference/Origin**
Agrobacterium tumefaciens			
JC2140 (named WT in this article)	C58	Wild-type *Agrobacterium tumefaciens* C58 with dicentric chromosome, pTiC58 and pAtC58.	(8)
JC2141 (named WT' in this article)	C58 *ΔtetRA*::a-*att*Tn*7*	Replacement of the *tetRA* locus with an artificial *att*Tn*7* site. Growth, motility and biofilm formation are identical to WT as shown in (8).	(8)
JC2291	WT' P*tac-ccrM*	P*tac-ccrM* inserted at the *ΔtetRA*::a-*att*Tn7 site (located between *ori2* and the *terR)* of the JC2141/WT' chromosome.	This study
JC2307	WT' *ΔccrM* P*tac-ccrM*		This study
JC2656	WT' PT7-*ygfp-parB*^{MT1}-*parS*^{MT1} inserted between *Atu_0047* and *Atu_0048*	PT7-*ygfp-parB*^{pMT1}-*parS*^{pMT1} reporter near *Atu_0048 (*50kbp away from *ori1)*	This study
JC2657	WT' PT7-*ygfp-parB*^{MT1}-*parS*^{MT1} inserted between *Atu_* 3973 and *Atu_3974*	PT7-*ygfp-parB*^{pMT1}-*parS*^{pMT1} reporter near *Atu_3973* (57kbp away from *ori2)*	This study
JC2777	WT' PT7-*mcherry-parB*^{P1}-*parS*^{P1} inserted between *Atu_0047* and *Atu_0048* and PT7-*ygfp-parB*^{MT1}-*parS*^{MT1} inserted between *Atu_* 3973 and *Atu_3974*	PT7-*mcherry-parB*^{P1}-*parS*^{P1} reporter near *Atu_0048 (*50kbp away from *ori1)* and PT7-*ygfp-parB*^{pMT1}-*parS*^{pMT1} reporter near *Atu_3973* (57kbp away from *ori2)*	This study
JC2660	WT' *ΔccrM* P*tac-ccrM* PT7-*ygfp-parB*^{MT1}-*parS*^{MT1} inserted between *Atu_0047* and *Atu_0048*	PT7-*ygfp-parB*^{pMT1}-*parS*^{pMT1} reporter near *Atu_0048 (*50kbp away from *ori1)*	This study
JC2661	WT' *ΔccrM* P*tac-ccrM* PT7-*ygfp-parB*^{MT1}-*parS*^{MT1} inserted between *Atu_* 3973 and *Atu_3974*	PT7-*ygfp-parB*^{pMT1}-*parS*^{pMT1} reporter near *Atu_3973* (57kbp away from *ori2)*	This study
JC2836	WT' *ΔccrM* P*tac-ccrM* PT7-*mcherry-parB*^{P1}-*parS*^{P1} inserted between *Atu_0047* and *Atu_0048* and PT7-*ygfp-parB*^{MT1}-*parS*^{MT1} inserted between *Atu_* 3973 and *Atu_3974*	PT7-*mcherry-parB*^{P1}-*parS*^{P1} reporter near *Atu_0048 (*50kbp away from *ori1)* and PT7-*ygfp-parB*^{pMT1}-*parS*^{pMT1} reporter near *Atu_3973* (57kbp away from *ori2)*	This study
JC2887	WT' P*tauA-gcrA*	P*tauA-gcrA* inserted at the *ΔtetRA*::a-*att*Tn7 site (located between *ori2* and the *terR)* of the JC2141/WT' chromosome.	This study
JC2899	WT' Δ*gcrA* P*tauA-gcrA*		This study

Escherichia coli			
TOP10		Used for cloning procedures and β-galactosidase assays	
JC1710	S17.1 λpir	Used for plasmid transfers from *E. coli* to *A. tumefaciens* (conjugation)	**(9)**
JC2916 = MFDpir	*pir+,* DAP auxotroph	Used to construct pSW25T derivatives and for conjugation assays with *S. meliloti* or DH5α	(10)
DH5α	*pir-*	Used for conjugation assays with MFDpir	
Sinorhizobium meliloti			
JC2707= Rm1021	Strep^R	Used for conjugation assays	(11)
Caulobacter crescentus			
JC450	Synchronizable CB15N wild-type strain (= NA1000)	Used for PCR amplification of *ccrM*^{Cc} and *rpoD*^{Cc} and for promoter activity assays	(12)

Table S2: Plasmids used in this study

**Plasmid name**	**Description**	**Reference/origin**
pTNS3	Helper plasmid encoding the site-specific TnsABCD Tn7 transposition pathway (Amp^R)	(8)
pUC-miniTn7TGMPtac-HA	Mini-Tn7 vector containing *lacIq* and *tac* promoter (Amp^R and Gm^R)	(8)
pUC-miniTn7TGMPtac-ccrM	*ccrM* ORF (*Atu_0794*) cloned into pUC-miniTn7TGMPtac-HA under the control of IPTG-inducible P*tac.*	This study
pNPTS138	Suicide vector with *sacB* gene and *oriT* (Km^R)	D. Alley, unpublished
pNPTS138-ΔccrM	pNPTS138-derived suicide vector to delete the *ccrM* gene in the *A. tumefaciens* genome	This study
pWX963	pNPTS138-derived suicide vector to integrate PT7-*ygfp-parB*^{MT1}*-parS*^{MT1} near *Atu_0048*	(13)
pWX967	pNPTS138-derived suicide vector to integrate PT7-*ygfp-parB*^{MT1}*-parS*^{MT1} near *Atu_3973*	(13)
pWX995	pNPTS138-derived suicide vector to integrate PT7-*mcherry-parB*^{P1}*-parS*^{P1} near *Atu_0048*	(14)
pRXMCS-2 (also named pRX in figures)	Low copy number vector with RK2 origin and *oriT* (Km^R)	(15)
pRX-P*tac-ccrM*	P*tac-ccrM* from pUC-miniTn7TGMPtac-ccrM cloned into pRXMCS-2	This study
pSW25T	Vector (Kan^R) with a unique conditional *oriV*R6Kγ origin of replication that can only replicate in *pir*+ bacterial strains. Also has an *oriT*$_{RP4}$ for conjugation	(16)
pSW25T-repABC^{Ch2}(WT)	pSW25T with the WT *repABC*^{Ch2}*/ori2* module	This study
pSW25T'	Derivative of pSW25T-repABC^{Ch2}(WT) with a deletion of most of the *repABC*^{Ch2} operon through a NdeI digestion	This study
pSW25T-repABC^{Ch2}(P$_A$-M1)	pSW25T with a mutant *repABC*^{Ch2}*/ori2* module: the M1 GANTC motif in P$_A$ is replaced by a GTNTC motif	This study

pSW25T-repABC^{Ch2}(P_A-M2)	pSW25T with a mutant *repABC^{Ch2}/ori2* module: the M2 GANTC motif in P_A is replaced by a GTNTC motif	This study
pSW25T-repABC^{Ch2}(P_A-M3)	pSW25T with a mutant *repABC^{Ch2}/ori2* module: the M3 GANTC motif in P_A is replaced by a GTNTC motif	This study
pSW25T-repABC^{Ch2}(P_E-M1)	pSW25T with a mutant *repABC^{Ch2}/ori2* module: the M1 GANTC motif in P_E is replaced by a GTNTC motif	This study
pSW25T-repABC^{Ch2}(P_E-M2)	pSW25T with a mutant *repABC^{Ch2}/ori2* module: the M2 GANTC motif in P_E is replaced by a GTNTC motif	This study
pSW25T-repABC^{Ch2}(P_E-M3)	pSW25T with a mutant *repABC^{Ch2}/ori2* module: the M3 GANTC motif in P_E is replaced by a GTNTC motif	This study
pOT1e	pBBR1 with promoter-less *egfp,* Gent^R	(17)
pP_A(WT)-egfp	pOT1e with a WT P_A promoter region controlling *egfp* expression	This study
pP_A(M1)-egfp	pOT1e with a mutant P_A promoter region controlling *egfp* expression: the M1 GANTC motif in P_A is replaced by a GTNTC motif	This study
pP_A(M2)-egfp	pOT1e with a mutant P_A promoter region controlling *egfp* expression: the M2 GANTC motif in P_A is replaced by a GTNTC motif	This study
pP_A(M3)-egfp	pOT1e with a mutant P_A promoter region controlling *egfp* expression: the M3 GANTC motif in P_A is replaced by a GTNTC motif	This study
pP_E(WT)-egfp	pOT1e with a WT P_A promoter region controlling *egfp* expression	This study
pP_E(M1)-egfp	pOT1e with a mutant P_A promoter region controlling *egfp* expression: the M1 GANTC motif in P_E is replaced by a GTNTC motif	This study
pP_E(M2)-egfp	pOT1e with a mutant P_A promoter region controlling *egfp* expression: the M2 GANTC motif in P_E is replaced by a GTNTC motif	This study
pP_E(M3)-egfp	pOT1e with a mutant P_A promoter region controlling *egfp* expression: the M3 GANTC motif in P_E is replaced by a GTNTC motif	This study
pUC-miniTn7TGMPtac-gcrA	*gcrA* ORF (*Atu_0426*) cloned into pUC-miniTn7TGMPtac-HA under the control of IPTG-inducible P*tac*	This study
pJC43	Vector carrying the *tauR* ORF and the P*tauA* promoter	(18)
pUC-miniTn7TGMPtauA-gcrA	*tauR-PtauA* cloned into pUC-miniTn7TGMPtac-gcrA instead of P*tac*. The expression of *gcrA* is now under the control of Taurine-inducible P*tauA*	This study
pNPTS138-ΔgcrA	pNPTS138-derived suicide vector to delete the *gcrA* gene in the *A. tumefaciens* genome	This study
p*lacZ*290	Low copy number vector with RK2 origin and *oriT,* used to create *lacZ* transcriptional fusions (Tet^R)	(19)
p*lacZ*290-P*gcrA*	*gcrA* promoter region (250 bp upstream of *gcrA* ORF) cloned into p*lacZ*290 and controlling *lacZ* transcription	This study
pBAD24-CcrM-WT	Vector encoding the *Caulobacter crescentus* CcrM protein from an arabinose-inducible promoter (Amp^R)	(20)
pBAD24-CcrM-mut	Vector encoding a catalytically inactive *Caulobacter crescentus* CcrM(D31A) mutant protein from an arabinose-inducible promoter (Amp^R)	(20)

pBAD-RpoD^{Cc-}CcrM^{Cc}-WT	*Caulobacter crescentus rpoD* ORF (*CCNA_03142*) cloned into pBAD24-CcrM-WT and controlled by arabinose-inducible promoter	This study
pBAD-RpoD^{Cc-}CcrM^{Cc}-mut	*Caulobacter crescentus rpoD* ORF (*CCNA_03142*) cloned into pBAD24-CcrM-mut and controlled by arabinose-inducible promoter	This study

Table S3: Oligonucleotides used in this study

**Primer name**	**Sequence (5'→3')**	**Used for:**
Primers used for cloning procedures and strain constructions:		
MS9	GTACATATGGCAGCAGTTTTTCCGCTGG	Construction of pUC-miniTn7TGMPtac-ccrM
MS10	CGAGATCTCTATTCAGCCTTTGCCATTTCAC	Construction of pUC-miniTn7TGMPtac-ccrM
Tet-Forward	ACATGTTGTATACCGGAAACTGATTGCAC	Verification of P*tac-ccrM* or P*tauA-gcrA* insertions into genome
Tn7R109	CAGCATAACTGGACTGATTTCAG	Verification of P*tac-ccrM* or P*tauA-gcrA* insertions into genome
MS1	GCCAAGCTTCAATTTTGCTTCGAAGGTGGCGT	Constructions of pNPTS138-ΔccrM and strain JC2307
MS2	TGCGGATCCTGGTACTCGCTCCATACGCTTAC	Construction of pNPTS138-ΔccrM
MS3	GCTGGATCCTCGAGAGCCGTAGAGGCTGGAG	Construction of pNPTS138-ΔccrM
MS4	GGGGCTAGCTACCGCTCCAGTCCTGCGCAAT	Constructions of pNPTS138-ΔccrM and strain JC2307
MS17	TCCTTCACCTCGAAGGGCAC	Construction of strain JC2307
MS40	CCAGAATTCGCTGTGGTATGGCTGTGCAG	Construction of pRX-P*tac-ccrM*
MS41	GGCAGATCTTAGCTAGCTCATTAAGCGTAATCTGGAAC	Construction of pRX-P*tac-ccrM*
FM15	GGAAAGAGTTTAAATAATAGGGCCGCTCTAGAACTAGTGG	Construction of pSW25T-repABC^{Ch2}(WT)
FM16	GGAAAGAGTTTAAATAATAGGGCCGCTCTAGAACTAGTGG	Construction of pSW25T-repABC^{Ch2}(WT)
JC309	CTATTATTTAAACTCTTTCCAGCCGTTGACGGCACGATTG	Construction of pSW25T-repABC^{Ch2}(WT)

JC310	TACGTAGAATGTATCAGACTAAAGATGGGCGGCGGTACAG	Construction of pSW25T-repABC^{Ch2}(WT)
ffjc102	CTCCAAGCATCATAAAGGCG	Construction of pSW25T-repABC^{Ch2}(P_A-M1)
ffjc103	CCGGCAGCCAGTCGAACCCG	Construction of pSW25T-repABC^{Ch2}(P_A-M1)
ffjc100	CATTGTGGAAGCATCACATTTG	Construction of pSW25T-repABC^{Ch2}(P_A-M2)
ffjc101	CTTTAGCGGCAGTTCTGCACC	Construction of pSW25T-repABC^{Ch2}(P_A-M2)
ffjc91	GCTTATATCTCAGTTGCGGAAAAAACC	Construction of pSW25T-repABC^{Ch2}(P_A-M3)
ffjc92	GCCGCTTCTCTTTTTGGCGC	Construction of pSW25T-repABC^{Ch2}(P_A-M3)
ffjc52	CTCTTGACTATGGTTCGCGGAAATG	Construction of pSW25T-repABC^{Ch2}(P_E-M1)
ffjc58	TCTTGGCATCGAATTTTTCGG	Construction of pSW25T-repABC^{Ch2}(P_E-M1)
ffjc50	CGATGCCAAGGCTCTTGACTATG	Construction of pSW25T-repABC^{Ch2}(P_E-M2)
ffjc57	AATTTTTCGGTGAGCGATTTTC	Construction of pSW25T-repABC^{Ch2}(P_E-M2)
ffjc106	CGCGAATCATAGTCAAGAGTCTTG	Construction of pSW25T-repABC^{Ch2}(P_E-M3)
ffjc107	GAAATGAGGTTCTTTGGTTGCTTAG	Construction of pSW25T-repABC^{Ch2}(P_E-M3)
ffjc113	GTACGAAGCTTGATGGCCGAGAAACCCGACC	Construction of all pP_A-egfp derivatives
ffjc114	GTACGACTAGTCGGTCTTCTTTCGCTCGAAATTG	Construction of all pP_A-egfp derivatives
ffjc115	GTACGAAGCTTGTCTTTCACCTTTCACAAGG	Construction of all pP_E-egfp derivatives
ffjc116	GTACGACTAGTAACCAAAGAATCTCATTTCCG	Construction of all pP_E-egfp derivatives
ffjc123	GTACGACTAGTAACCAAAGAACCTCATTTCCG	Construction of pP_E(M3)-egfp

MS38	CCGAGATCTTCAAGCCTTACGGCGCTCGCTAACCGG	Construction of pUC-miniTn7TGMPtac-gcrA
MS39	AGCCATATGAACTGGACAGACGAGCG	Construction of pUC-miniTn7TGMPtac-gcrA
MS167	CTGTTCGAATTTCTGCCATTCATCCGC	Construction of pUC-miniTn7TGMPtauA-gcrA
MS168	CCACATATGTGAATTCCTCCTGCTAGAGTCG	Construction of pUC-miniTn7TGMPtauA-gcrA
MS5	CCAGGATCCCTCGCCTCGCTCAAAGCGCAG	Construction of pNPTS138-ΔgcrA
MS6	CCAGCTAGCGCCGCTTCTCCGCTTTTCGCG	Construction of pNPTS138-ΔgcrA
MS7	AAGGCTAGCGTGTTGCGGCACGCATTTGACA	Construction of pNPTS138-ΔgcrA
MS8	CGCATGCAATTCCGCCTGCTGGAGTTC	Construction of pNPTS138-ΔgcrA
MS16	GCCGGCCGCAAAACGAGAAA	Construction of strain JC2899
MS46b	CCACTGCAGGCCGCTTCTCCGCTTTTC	Construction of p*lacZ*290-P*gcrA*
MS47	TGGGAATTCCTTTCGAGGCCCAAGGTG	Construction of p*lacZ*290-P*gcrA*
LC26	GCGCAAGCTTAGGAGGAATTCACCATGAGCAACAATTCCTCGGCCGA	Construction of pBAD-RpoD^{Cc}-CcrM^{Cc}-WT and pBAD-RpoD^{Cc}-CcrM^{Cc}-mut
LC29	AAAAAAAAGCTTTTACGAGTCCAGGAAGCTGCGCA	Construction of pBAD-RpoD^{Cc}-CcrM^{Cc}-WT and pBAD-RpoD^{Cc}-CcrM^{Cc}-mut
LC32	GCGAGGACCTCTCGGACGCCG	Construction of pBAD-RpoD^{Cc}-CcrM^{Cc}-WT and pBAD-RpoD^{Cc}-CcrM^{Cc}-mut
LC33	GCGGATGTCGGTGACCGACTGG	Construction of pBAD-RpoD^{Cc}-CcrM^{Cc}-WT and pBAD-RpoD^{Cc}-CcrM^{Cc}-mut
Primers used for qRT-PCR:		
MS90	CGCTTCGTGCAGAAGGACT	*hemF*
MS91	GCACGCCGACCTTTTCGAA	
MS80	GCGCTCTGATGCGGGTTTT	*purH*
MS81	AACTGGTTTGCCGAAGCACTG	
MS84	CTGCCGTCCTTGCCGGAAA	*yidC*
MS85	AGCCGGTAATTCCCCCTGTATT	
MS154	GGACTGGCTGTTCCCGATTT	*ccrM*
MS155	CTGTGTGGGATGCACCTTCTT	
MS100	GCCGACGCTCGTCGTCGTAA	*repA^{Ch2}*
MS101	CGATCCCCAGGCCAGTCT	

MS102	GGGAACCGCATCTGCAGTTT	*repB*^{Ch2}
MS103	GTCGCCCAGGGTCAGGAA	
MS104	GCGGGCATAACGTTTTCCGTT	*repC*^{Ch2}
MS105	GGCATGGCCGAACAGACATT	
MS98c	AGTTCCGTCAGCGTCAGG	*gcrA*
MS99b	GCGTCGAGACTGCAGAAGTTC	
MS146	GGAGCGTGGTAGACCGGT	*ftsZ*^{AT}
MS147	CAGCAGCCTTCTCAGGACTT	
MS110	CCGCAGGCTAGTCTTTCAGCA	*repA*^{pTi}
MS111	CGGTCGGATCACATCTCGCAA	
MS112	GGCTGCCGGTCGATATCAGAT	*repB*^{pTi}
MS113	AGGTCAGCGCGGTTAAGATTT	
MS114	GGTGCCGAACTGGTCGTAT	*repC*^{pTi}
MS115	GAGCCCGCAATCAACCAGTAT	
MS118	CGGTGCAAACGAGACGATTTA	*repA*^{pAt}
MS119	GCCCGGAATAAGGTCGATACCAT	
MS120	GCAGCCAAGGATCTCGGAATT	*repB*^{pAt}
MS121	GCGCGCTCGATCCAGGTTA	
MS122b	GGCAAGTCGGCAGACAAATGGA	*repC*^{pAt}
MS123b	CGCAGTTCGTTCTCGGGATAAA	
Primers used to test if the C58 derivatives (WT/WT') are fusion strains with a dicentric chromosome		
F1	TGTCTGGGTTCTGGAATTCGACGC	(1)
R1	AGGTTCCGTGGTATAGTTGTAGGC	(1)
F2	CTTGATCCAGAGTGATTTCGACGC	(1)
R2	CCTTGTGAACAACGCCTTTGACCC	(1)

Table S4 (Excel Supplementary file): RNA-Seq results comparing the transcriptome of JC2307 (*ΔccrM* P*tac-ccrM*) cells cultivated in ATGN +/- IPTG (7 hours) and of JC2140 (WT) cells cultivated in ATGN-IPTG. Replicon NC_003062.2 corresponds to Ch1 in non-fusion strains, replicon NC_003063.2 corresponds to Ch2 in non-fusion strains, replicon NC_003064.2 corresponds to pAt and replicon NC_003065.2 corresponds to pTi. Activated (FC>2) genes are highlighted in green. Repressed (FC>2) genes are highlighted in red. Adjusted P-values >0.01 (non-significant changes) are highlighted in grey. Genes of particular interest that are discussed in this study are highlighted in yellow. For COG annotations, the COG table in json format for *Agrobacterium tumefaciens* (Agrobacterium_fabrum_C58) was downloaded from the NCBI Database of Clusters of Orthologous Genes (https://www.ncbi.nlm.nih.gov/research/cog/) (21). The COG json table was reformatted into text format and gene identifiers were matched to COG terms via a customised Perl script. For missing terms, the NIH GenBank database of *Agrobacterium tumefaciens* protein sequences was used (22). GANTC motifs in 200 bp sequences upstream of the annotated start codon of each gene were searched using an ad hoc Perl script to find motif matches in FASTA-formatted sequences.

Table S5 (Excel Supplementary file): Lists of genes that were significantly mis-regulated (FC>2 and adjusted P-value<0.01) when comparing the transcriptome of JC2307 (*ΔccrM* P*tac-ccrM*) cells cultivated in ATGN +/- IPTG (7 hours) and genes that can be considered as part of the "direct regulon" of CcrM (FC>2, adjusted P-value<0.01 and minimum one GANTC motif in the 200 bp upstream of ORF). Activated genes are highlighted in green. Repressed genes are highlighted in red. Genes of particular interest that are discussed in this study are highlighted in yellow. In the "direct regulon" tab, orthologous genes that also belong to the "direct regulon" of the *Caulobacter crescentus* NA1000 CcrM protein (from (23)) are highlighted in blue and orthologous genes that also belong to the "direct regulon" of the *Brevundimonas subvibrioides* CcrM protein (from (24)) are highlighted in orange.

To search for genes orthologous to genes that belong to the "direct regulon" of CcrM in *A. tumefaciens* in the *C. crescentus NA1000* (NC_011916.1) or *B. subvibrioides* (NC_014375.1) genomes, the *blastP* alignment program (25) was used. In short, blast-formatted sequence databases were created for the three genomes and a blastP search was performed in command-line mode, with the following parameter options: "-num_alignments 5 -subject_besthit -outfmt "6 qacc qlen qstart qend sacc slen sstart send length pident evalue bitscore score mismatch gapopen gaps stitle". For further validation and complementary information, *blastP* searches were supplemented with searches based on COG identifiers between genes in the different species. This analysis revealed that only 4 genes belonging to the *C. crescentus* "direct" CcrM regulon also belonged to the *A. tumefaciens* "direct" CcrM regulon (1st blatsp matches and COG clusters). Similarly, only two genes belonging to the *B. subvibrioides* "direct" CcrM regulon also belonged to the *A. tumefaciens* "direct" CcrM regulon (2nd/3rd best blastp matches).

SUPPLEMENTARY METHODS:

Bacterial growth conditions

E. coli strains were grown using standard growth conditions in/on Luria-Bertani (LB: 1% tryptone, 0.5% yeast extract, 1% NaCl, pH 7) +/- bacto-agar (1.5%) medium at 37°C. Antibiotics were added at the following concentrations when needed (liquid/solid media): kanamycin 30/50 µg/mL, oxy-tetracycline 12/12 µg/mL, ampicillin 50/100 µg/mL. When indicated, diaminopimelic acid (DAP) was added at 0.3 mM.
A. tumefaciens strains were grown at 28°C in LB, Yeast Extract Beef (26) (YEB recipe from fisher scientific Bioworld 306270311: 0.5% tryptone, 0.1% yeast extract, 0.5% nutrient broth powder, 0.5% sucrose, 0.049% $MgSO_4$-$7H_2O$, pH 7.2) or AT minimal medium (without exogenous iron) (4) with 0.5% glucose (ATGN) or 5% sucrose (ATSN). Agar plates and soft-agar plates were prepared with 1.35% (ATGN), 1.2% (ATSN) or 0.25% (soft) bacto-agar, respectively. Antibiotics were added at the following concentrations when needed (liquid/solid media): kanamycin 150/300 µg/mL in all media, gentamycin 100/200 in ATGN/ATSN, oxy-tetracycline 3/3 µg/mL in ATGN. When indicated, IPTG or Taurine were added at a final concentration of 1mM (liquid/solid media) and 2.5/20mM (liquid/solid media), respectively.

S. meliloti strains were grown at 30°C in/on LB supplemented or not with bacto-agar 1.5%. When indicated, streptomycin (400 µg/ml) and/or kanamycin (50 µg/ml) were added.

C. crescentus strains were grown at 28°C in/on peptone yeast extract (PYE) or M2G minimal media (27), supplemented or not with bacto-agar 1.5%. When indicated, gentamicin was added to PYE medium at the following concentrations: 1µg/ml for liquid and 5 µg/ml for solid media. In M2G liquid medium, gentamicin was added at 40 µg/ml.

Construction of plasmids and strains

Plasmids (Table S2) were constructed using standard DNA cloning techniques and the inserts of all constructs were verified by Sanger sequencing (Microsynth AG or Sigma AG). The sequence of primers used during cloning procedures is described in Table S3. *E. coli* transformations were carried out using standard methods. When necessary, replicating or integrative plasmids were introduced into *A. tumefaciens* cells by conjugation or electroporation (8). *E. coli* S17.1 *λpir*/MFD*pir* or TOP10 strains were used for conjugation and cloning procedures, respectively.

- Construction of pUC-miniTn7TGMPtac-ccrM: The *ccrM* (*Atu0794*) ORF of the *A. tumefaciens* C58 strain was amplified from gDNA using primers MS9 and MS10, digested with NdeI/BglII and ligated into NdeI/BamHI-digested pUC-miniTn7TGMPtac-HA.
- Construction of pNPTS138-ΔccrM: The 500 bp sequences upstream and downstream of the *A. tumefaciens* C58 *ccrM* ORF were amplified using primer pairs MS1/MS2 and MS3/MS4, and digested by HindIII/BamHI and BamHI/NheI, respectively. Both fragments were then ligated into HindIII/NheI-digested pNPTS138.
- Construction of pRX-P*tac-ccrM:* The P*tac-ccrM* construct was amplified from pUC-miniTn7TGMPtac-ccrM using primers MS40 and MS41, digested by EcoRI/BglII and cloned into EcoRI/BglII-digested pRXMCS-2.
- Construction of the JC2291 strain: The pUC-miniTn7TGMPtac-ccrM plasmid was introduced into the JC2141 strain by electroporation with the pTNS3 helper plasmid. P*tac-ccrM* was inserted at the *ΔtetRA*::a-*att*Tn7 site located between *ori2* and the *terR* on the linear chromosome of JC2141 (position ~4.2Mbp in Fig.1b), giving the JC2291 strain. This insertion was verified using primers Tet-Forward and Tn7R109 as described before (8).
- Construction of the JC2307 strain: The pNPTS138-ΔccrM plasmid was introduced into the JC2291 strain by conjugation (kanamycin-resistant *A. tumefaciens* colonies were selected on ATGN+IPTG as *E. coli* S17.1 λpir does not grow on ATGN (28)). Integration at the native *ccrM* locus of the JC2291 chromosome by homologous recombination was verified by checking sucrose-sensitivity and by colony-PCR using primers MS4 and MS17. The resulting strain was then cultivated over-night in ATGN+IPTG medium (without antibiotics) before plating onto ATSN+IPTG medium (plasmid re-excision step). Kanamycin-sensitive and sucrose-resistant colonies were then selected onto ATSN+IPTG+/-kanamycin plates. We then screened for Δ*ccrM* colonies by colony PCR using primers MS1 and MS4.
- Construction of JC2656/JC2657/JC2777/JC2660/JC2661 strains: pWX963 or pWX967 were introduced into JC2141 or JC2307 by conjugation using a kanamycin-resistance selection on ATGN+IPTG.
- Construction of JC2277/JC2836 strains: JC2657 and JC2661 were cultivated over-night in ATGN+/-IPTG medium (without antibiotics, for pWX963 re-excision step) before plating onto ATSN+/-IPTG medium. Kanamycin-sensitive and sucrose-resistant colonies were then selected onto ATGN+/-IPTG. We then screened for colonies that kept the *ori2/ygfp* reporter by fluorescence microscopy. The pWX995 plasmid (donor of *ori1/mcherry* reporter) was then introduced into these two kanamycin-sensitive by conjugation using a kanamycin-resistance selection on ATGN+IPTG.
- Construction of pSW25T-repABC^{Ch2} plasmids: The WT *repABC*^{Ch2} operons (as shown in Fig.6a) of the *A. tumefaciens* C58 strain was amplified from gDNA and assembled into pSW25T to create the pSW25T-repABC^{Ch2}(WT). Most of the *repABC*^{Ch2} operon was deleted from pSW25T-repABC^{Ch2}(WT) by NdeI restriction, giving the nearly empty pSW25T' control vector. pSW25T-repABC^{Ch2} derivatives were constructed by site-directed mutagenesis PCR using primers listed in Table S3 and the pSW25T-repABC^{Ch2}(WT) template.
- Construction of pP$_A$-egfp derivatives: The WT or mutant 479 bp P$_A$ promoter regions upstream of the *repA*^{Ch2} ORF (as shown in Fig.6a) of the *A. tumefaciens* C58 strain were amplified from pSW25T-repABC^{Ch2} derivatives using primers ffjc113/ffjc114, digested by HindIII/SpeI and cloned into HindIII/SpeI-digested pOT1e to create all the pP$_A$-egfp derivatives.
- Construction of pP$_E$-egfp derivatives: The WT or mutant 96 bp P$_E$ promoter regions (as shown in Fig.6a) of the *A. tumefaciens* C58 strain were amplified from pSW25T-repABC^{Ch2} derivatives using primers ffjc115/ffjc116 (or ffjc123 instead of ffjc116 for P$_E$(M3)), digested by HindIII/SpeI and cloned into HindIII/SpeI-digested pOT1e to create all the pP$_E$-egfp derivatives.
- Construction of pUC-miniTn7TGMPtac-gcrA: The *gcrA* (*Atu0426*) ORF of the *A. tumefaciens* C58 strain was amplified using primers MS38 and MS39, digested with NdeI/BglII and ligated into NdeI/BamHI-digested pUC-miniTn7TGMPtac-HA.

- Construction of pUC-miniTn7TGMPtauA-gcrA: The *tauR*-P*tauA* module of pJC43 was amplified using primers MS167 and MS168, digested with NdeI/BstBI and ligated into NdeI/AccI-digested pUC-miniTn7TGMPtac-gcrA, leading to a replacement of P*tac* by the *tauR*-P*tauA* module.
- Construction of the JC2887 strain: The pUC-miniTn7TGMPtauA-gcrA plasmid was introduced into the JC2141 strain by electroporation with the pTNS3 helper plasmid. The *tauR*-P*tauA-gcrA* construct was inserted at the *ΔtetRA*::a-*att*Tn7 site located between *ori2* and the *terR* on the linear chromosome of JC2141 (position ~4.2Mbp in Fig.1b). Insertion into the JC2887 genome was verified using primers Tet-Forward and Tn7R109 as described before (8).
- Construction of pNPTS138-ΔgcrA: The 500 bp sequences upstream and downstream of the *A. tumefaciens* C58 *gcrA* ORF were amplified from gDNA using primer pairs MS5/MS6 and MS7/MS8 and then digested by NheI/BamHI and BamHI/SphI, respectively. Both fragments were then ligated into SphI/NheI-digested pNPTS138.
- Construction of the JC2899 strain: The pNPTS138-ΔgcrA plasmid was introduced into the JC2887 strain by conjugation (kanamycin-resistant *A. tumefaciens* colonies were selected on ATGN +Taurine. Integration of the pNPTS138-*ΔgcrA* plasmid at the native *gcrA* locus by homologous recombination was verified by checking sucrose sensitivity and by colony-PCR using primers MS8 and MS16. The resulting strain was then cultivated overnight in ATGN +Taurine (without antibiotics) before plating onto ATSN +Taurine (plasmid re-excision step). Kanamycin-sensitive and sucrose-resistant colonies were then selected onto ATSN +Taurine +/-kanamycin plates. We then screened for Δ*gcrA* colonies by colony PCR using primers MS5 and MS8.
- Construction of p*lacZ*290-P*gcrA:* The 250 bp promoter region upstream of the *gcrA* (*Atu0426*) ORF of the *A. tumefaciens* C58 strain (JC2140) was amplified using primers MS46b and MS47, digested with PstI/EcoRI and ligated into PstI/EcoRI-digested p*lacZ*290.
- Constructions of pBAD-RpoD^{Cc}-CcrM^{Cc}-WT and pBAD-RpoD^{Cc}-CcrM^{Cc}-mut: The *rpoD^{Cc}* gene (*CCNA_03142)* was amplified from *C. crescentus* NA1000 gDNA using primers LC26 and LC29, creating a 5'AGGAGG3' *E. coli* ribosome binding site in frame with *rpoD^{Cc}*. The PCR product and the pBAD-RpoD^{Cc}-CcrM^{Cc}-WT or pBAD-RpoD^{Cc}-CcrM^{Cc}-mut vectors were then digested with the HindIII enzyme and ligated together. The insert sequence and orientation were checked using primers LC32 and LC33.

Biofilm/surface attachment assays

Biofilm assays were conducted as described previously (4) with minor adaptations. Briefly, 4 times 3mL of each culture diluted to an OD_{600}~0.05 were introduced into 4 wells containing a size-adjusted, vertical polyvinyl chloride (PVC) coverslip. 12-well polystyrene plates were incubated at room-temperature for 72 hours into a sealed container containing a smaller recipient with 200mL of saturated sulfate solution (120g/L). After incubation, the coverslips were removed from wells and immersed into deionized H_2O to remove planktonic cells. Dried coverslips were then immersed into 1mL of a 0.1% crystal violet (CV) solution for 5 minutes prior to rinsing with deionized H_2O and careful drying. For each assay, one coverslip was kept for imaging, while the other three were each immersed into 1mL of a 33% acetic acid solution. The A_{600} of the solubilized CV and the OD_{600} of the cultures from each well were then measured. The biofilm score was calculated as the A_{600} divided by the OD_{600}.

β-galactosidase assays

β-galactosidase assays were adapted from (28). Briefly, the OD_{600nm} of each culture was measured and a given volume (V, in mL and below 500μL) of each culture was collected. Z-buffer (without β-mercaptoethanol) was then added to each culture sample to reach a final volume of 1mL. 30μL of 0.05% SDS and 60μL chloroform were then added into these samples, which were then mixed vigorously. Samples were then incubated at 28°C for 5 minutes to reach optimum temperature. The β-galactosidase reaction was then started when 200μL of

ONPG at 4mg/mL was added. After a given reaction time (t, in minutes), the reaction was stopped with the addition of 500µL of 1M Na_2CO_3 and samples were briefly centrifuged before measuring the A_{420nm}. Miller Units (MU) were calculated using the following formula: MU = $1000*A_{420}/(OD_{600}*t*V)$. 3 biological replicates were used for each strain/condition.

SUPPLEMENTARY REFERENCES

1. Liao, Q., Ren, Z., Wiesler, E.E., Fuqua, C. and Wang, X. (2022) A dicentric bacterial chromosome requires XerC/D site-specific recombinases for resolution. *Curr Biol*, **32**, 3609-3618 e3607.
2. Chai, T., Terrettaz, C. and Collier, J. (2021) Spatial coupling between DNA replication and mismatch repair in *Caulobacter crescentus*. *Nucleic Acids Res*, **49**, 3308-3321.
3. Schindelin, J., Arganda-Carreras, I., Frise, E., Kaynig, V., Longair, M., Pietzsch, T., Preibisch, S., Rueden, C., Saalfeld, S., Schmid, B. *et al.* (2012) Fiji: an open-source platform for biological-image analysis. *Nat Methods*, **9**, 676-682.
4. Morton, E.R. and Fuqua, C. (2012) Laboratory maintenance of *Agrobacterium*. *Curr Protoc Microbiol*, **Chapter 1**, Unit3D 1.
5. Izquierdo, J., Venkova-Canova, T., Ramirez-Romero, M.A., Tellez-Sosa, J., Hernandez-Lucas, I., Sanjuan, J. and Cevallos, M.A. (2005) An antisense RNA plays a central role in the replication control of a repC plasmid. *Plasmid*, **54**, 259-277.
6. Chai, Y. and Winans, S.C. (2005) A small antisense RNA downregulates expression of an essential replicase protein of an *Agrobacterium tumefaciens* Ti plasmid. *Mol Microbiol*, **56**, 1574-1585.
7. Park, J., Taslim, C. and Lin, S. (2015) BOG: R-package for Bacterium and virus analysis of Orthologous Groups. *Comput Struct Biotechnol J*, **13**, 366-369.
8. Figueroa-Cuilan, W., Daniel, J.J., Howell, M., Sulaiman, A. and Brown, P.J. (2016) Mini-Tn7 Insertion in an Artificial attTn7 Site Enables Depletion of the Essential Master Regulator CtrA in the Phytopathogen *Agrobacterium tumefaciens*. *Appl Environ Microbiol*, **82**, 5015-5025.
9. Simon R, P.U., Puhler A. (1983) A broad host range mobilization system for *in vivo* genetic engeneering: Transposon mutagenesis in gram negative bacteria. *Bio/Technology*, **1**, 784-790.
10. Ferrieres, L., Hemery, G., Nham, T., Guerout, A.M., Mazel, D., Beloin, C. and Ghigo, J.M. (2010) Silent mischief: bacteriophage Mu insertions contaminate products of *Escherichia coli* random mutagenesis performed using suicidal transposon delivery plasmids mobilized by broad-host-range RP4 conjugative machinery. *J Bacteriol*, **192**, 6418-6427.
11. Meade, H.M., Long, S.R., Ruvkun, G.B., Brown, S.E. and Ausubel, F.M. (1982) Physical and genetic characterization of symbiotic and auxotrophic mutants of *Rhizobium meliloti* induced by transposon Tn5 mutagenesis. *J Bacteriol*, **149**, 114-122.
12. Evinger, M. and Agabian, N. (1977) Envelope-associated nucleoid from *Caulobacter crescentus* stalked and swarmer cells. *J Bacteriol*, **132**, 294-301.
13. Ren, Z., Liao, Q., Karaboja, X., Barton, I.S., Schantz, E.G., Mejia-Santana, A., Fuqua, C. and Wang, X. (2022) Conformation and dynamic interactions of the multipartite genome in *Agrobacterium tumefaciens*. *Proc Natl Acad Sci U S A*, **119**.
14. Ren, Z., Liao, Q., Barton, I.S., Wiesler, E.E., Fuqua, C. and Wang, X. (2022) Centromere Interactions Promote the Maintenance of the Multipartite Genome in *Agrobacterium tumefaciens*. *mBio*, **13**, e0050822.

15. Thanbichler, M., Iniesta, A.A. and Shapiro, L. (2007) A comprehensive set of plasmids for vanillate- and xylose-inducible gene expression in *Caulobacter crescentus*. *Nucleic Acids Res*, **35**, e137.
16. Demarre, G., Guerout, A.M., Matsumoto-Mashimo, C., Rowe-Magnus, D.A., Marliere, P. and Mazel, D. (2005) A new family of mobilizable suicide plasmids based on broad host range R388 plasmid (IncW) and RP4 plasmid (IncPalpha) conjugative machineries and their cognate *Escherichia coli* host strains. *Res Microbiol*, **156**, 245-255.
17. Pothier, J.F., Wisniewski-Dye, F., Weiss-Gayet, M., Moenne-Loccoz, Y. and Prigent-Combaret, C. (2007) Promoter-trap identification of wheat seed extract-induced genes in the plant-growth-promoting rhizobacterium *Azospirillum brasilense* Sp245. *Microbiology (Reading)*, **153**, 3608-3622.
18. Czarnecki, J., Chapkauskaitse, E., Bos, J., Sentkowska, D., Wawrzyniak, P., Wyszynska, A., Szuplewska, M. and Bartosik, D. (2022) Differential Localization and Functional Specialization of *parS* Centromere-Like Sites in *repABC* Replicons of *Alphaproteobacteria*. *Appl Environ Microbiol*, **88**, e0020722.
19. Gober, J.W. and Shapiro, L. (1992) A developmentally regulated *Caulobacter* flagellar promoter is activated by 3' enhancer and IHF binding elements. *Mol Biol Cell*, **3**, 913-926.
20. Maier, J.A.H., Mohrle, R. and Jeltsch, A. (2017) Design of synthetic epigenetic circuits featuring memory effects and reversible switching based on DNA methylation. *Nat Commun*, **8**, 15336.
21. Galperin, M.Y., Makarova, K.S., Wolf, Y.I. and Koonin, E.V. (2015) Expanded microbial genome coverage and improved protein family annotation in the COG database. *Nucleic Acids Res*, **43**, D261-269.
22. Jack, D.L., Yang, N.M. and Saier, M.H., Jr. (2001) The drug/metabolite transporter superfamily. *Eur J Biochem*, **268**, 3620-3639.
23. Gonzalez, D., Kozdon, J.B., McAdams, H.H., Shapiro, L. and Collier, J. (2014) The functions of DNA methylation by CcrM in *Caulobacter crescentus*: a global approach. *Nucleic Acids Res*, **42**, 3720-3735.
24. Adhikari, S., Erill, I. and Curtis, P.D. (2021) Transcriptional rewiring of the GcrA/CcrM bacterial epigenetic regulatory system in closely related bacteria. *PLoS Genet*, **17**, e1009433.
25. Altschul, S.F., Gish, W., Miller, W., Myers, E.W. and Lipman, D.J. (1990) Basic local alignment search tool. *J Mol Biol*, **215**, 403-410.
26. Holsters, M., de Waele, D., Depicker, A., Messens, E., van Montagu, M. and Schell, J. (1978) Transfection and transformation of *Agrobacterium tumefaciens*. *Mol Gen Genet*, **163**, 181-187.
27. Ely, B. (1991) Genetics of *Caulobacter crescentus. Methods Enzymol*, **204**, 372-384.
28. Morton, E.R. and Fuqua, C. (2012) Genetic manipulation of *Agrobacterium*. *Curr Protoc Microbiol*, **Chapter 3**, Unit 3D 2.
