# Supplementary material for: DNA methylation by CcrM contributes to genome maintenance in the Agrobacterium tumefaciens plant pathogen
Source: Nucleic Acids Res. 2024 Sep 4;52(19):11519–35. doi: 10.1093/nar/gkae757 (PMC11514494; doi:10.1093/nar/gkae757)
Supplement: gkae757_Supplemental_Files [file gkae757_supplemental_files.zip › GlimmaVolcanoPlot.html.zip!/GlimmaVolcanoPlot.html]

glimmaXY
